# Tuning metal segregation and surface composition of Pt–Pd nanoparticles in microemulsions: the role of nucleation kinetics

**DOI:** 10.1039/d6ra06921e

**Published:** 2026-09-29

**Authors:** Concha Tojo

**Affiliations:** a Department of Physical Chemistry, University of Vigo E-36310 Vigo Spain ctojo@uvigo.es

## Abstract

A kinetic simulation model was used to investigate the formation of Pt–Pd nanoparticles synthesized in microemulsions by the one-pot method and to identify the mechanisms governing metal segregation and surface composition. Metal segregation is determined by the interplay between reduction, nucleation, reactant concentration, and intermicellar exchange. High reactant concentrations increase the availability of metal atoms, allowing the nucleation requirements to be satisfied earlier. This promotes Pt incorporation during the initial stages of nanoparticle formation, favouring Pt-core/Pd-shell structures. In contrast, low reactant concentrations produce alloyed or partially segregated nanoparticles. Surfactant film flexibility mainly affects the surface composition by modulating the contribution of Ostwald ripening to particle growth, with higher flexibility promoting Pt surface enrichment. The critical nucleus size for heteroatomic nucleation is identified as the dominant nucleation parameter governing the degree of metal segregation. The model successfully rationalizes experimental results exhibiting markedly different atomic arrangements and provides mechanistic insight into Pt–Pd nanoparticle formation in microemulsions. These findings provide practical guidelines for tailoring the internal architecture and surface composition of Pt–Pd nanoparticles through the appropriate selection of synthesis conditions.

## Abstract 2

A kinetic simulation model was used to investigate the formation of Pt–Pd nanoparticles synthesized in microemulsions by the one-pot method and to identify the mechanisms governing metal segregation and surface composition.

## Graphical abstract

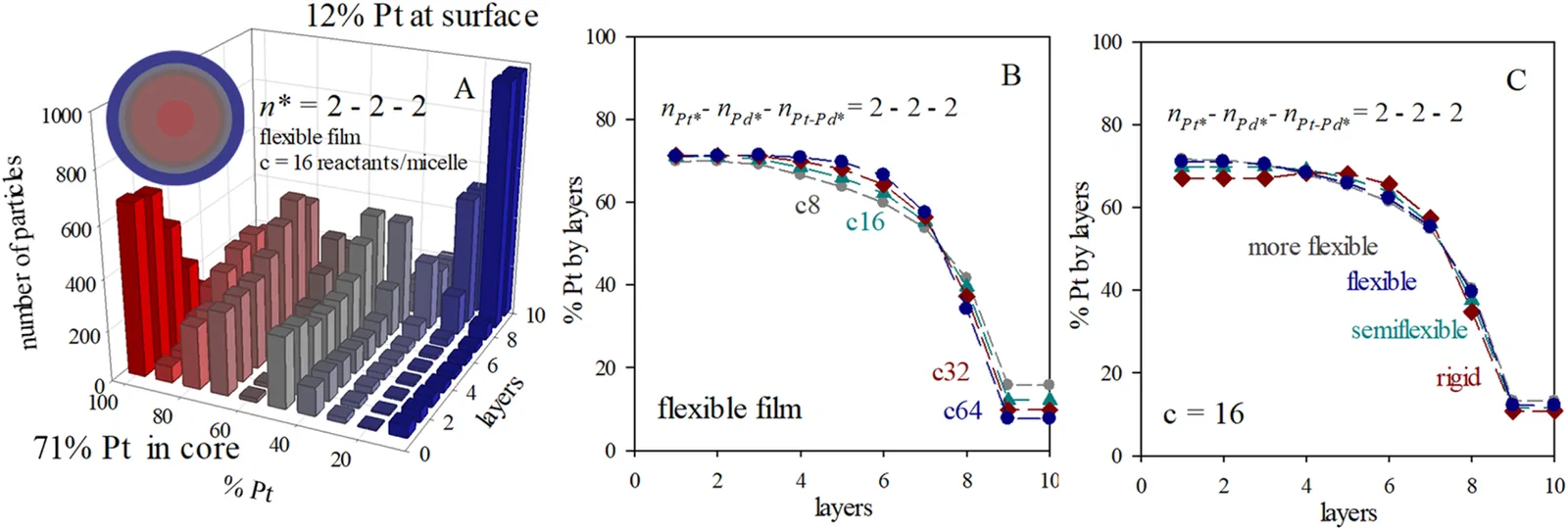

## 1 Introduction

Platinum is among the most effective catalysts for electrochemical processes. However, its high cost and scarcity drive the development of strategies to minimize Pt loading. Alloying Pt with other metals has emerged as a particularly effective approach, enabling lower Pt consumption while preserving or even enhancing catalytic performance. In addition, nanoalloys offer the possibility of tailoring physicochemical properties through the synergistic combination of two metals and precise nanoscale control over particle size, composition, and atomic ordering. Although Pd and Pt are both intrinsically active catalysts, their catalytic activity and selectivity can be further improved through alloying. In particular, Pt/Pd nanoparticles exhibit enhanced catalytic activity across a wide range of reactions compared with their monometallic counterparts.^1–3^ Consequently, the Pt/Pd bimetallic system was selected as a case study owing to its remarkable catalytic performance in a number of chemical applications.^3–7^

Because the adsorption and desorption of species occur at the nanocatalyst surface, catalytic performance is highly sensitive to the surface composition and atomic distribution of the constituent elements within the outermost layers of bimetallic catalysts. However, achieving precise control over the arrangement of both metals, particularly in the near-surface layers, remains a major challenge. Further advances in the catalytic performance therefore depend on the ability to synthesize materials with well-defined surface composition.^8–11^ Notably, the surface composition of alloy nanoparticles often differs from that of the bulk, with important consequences for their catalytic properties. The lower surface energy of Pd (1.43 J m^−2^)^12^ compared with Pt (1.5–2.03 J m^−2^)^13^ favors Pd surface segregation, while the higher heat of sublimation of Pt (565.5 kJ mol^−1^)^14^ than Pd (381 kJ mol^−1^),^12^ indicative of stronger metal–metal interactions, promotes Pt enrichment in the nanoparticle core. By contrast, the nearly identical atomic sizes of Pt and Pd (lattice mismatch ∼0.8%) suggest that strain-driven contributions are minimal. Finally, kinetic aspects must also be considered. During the simultaneous reduction of Pt and Pd precursors, differences in reduction rates can significantly influence nanoparticle structure. Because Pt has a higher redox potential, it is typically reduced first, promoting the formation of Pt-rich nuclei. The more slowly reduced Pd can subsequently deposit onto these nuclei, favoring the formation of nanoparticles with a Pt-enriched core and a Pd-enriched surface. Consistent with this expectation, Pd surface segregation in Pt/Pd nanoparticles has been reported experimentally.^3,15^ This observation is further supported by theoretical calculations, which predict that segregation is energetically favored in Pt/Pd systems, leading to Pt enrichment in the nanoparticle core and Pd enrichment at the surface.^16–19^ Pd-core/Pt-shell configurations have been identified as the least structurally stable among the possible arrangements.^20^ Finally, molecular dynamics simulations have revealed partial Pd–Pt intermixing within the core of onion-like shell clusters, though Pd enrichment of the surface is preserved.^21^

Finally, the final nanostructure is strongly influenced by the synthesis method. In fact, even the inverse Pd-core/Pt-shell architecture has been successfully synthesized *via* a sequential addition approach, in which the Pd core is first formed, and Pt ions are subsequently added, reduced, and deposited onto the preformed Pd core to build the shell.^22,23^ These results highlight that precise control of the surface composition requires a detailed understanding of the specific synthetic route. In this work, we focus on the microemulsion method, a widely used and versatile approach for nanoparticle synthesis. This method enables the preparation of bimetallic nanoparticles across a broad compositional range, including pairs that exhibit a bulk miscibility gap (see, for example, Au–Rh,^24^ Au–Pt,^25,26^ Pt–Rh,^26^ Pd–Rh^26^). Moreover, Pt/Pd nanoparticles prepared by the microemulsion method have been reported to exhibit higher catalytic activity than those synthesized by other routes.^3^ However, even within this approach, distinct nanostructures can be obtained. In fact, Pd/Pt nanoparticles have been reported as homogeneous alloys,^27,28^ as core–shell nanoparticles,^29^ as non-core–shell particles with Pd surface enrichment,^3^ and even as structures with Pt-enriched surfaces.^4,30^

To understand the diversity of nanostructures obtained, the complexity of the reaction medium must be considered. Water-in-oil microemulsion consist of reverse micelles, *i.e.*, water droplets dispersed in a continuous oil phase and stabilized by surfactant molecules at the oil–water interface. Metal precursors and the reducing agent are confined within these aqueous nanodomains, where micelles act as nanoreactors for reduction, nucleation and growth processes under spatial confinement. Material exchange between micelles occurs through transient micelle–micelle fusion and redispersion events, which arise from collisions that temporarily disrupt the surfactant film and generate short-lived intermicellar channels for species transfer. Consequently, intermicellar exchange governs the encounter between reducing agents and metal precursors and therefore controls reduction, nucleation, growth, and ultimately the atomic arrangement within the nanoparticles. In addition, the final nanoarchitecture also depends on the mixing pattern of reactants, the specific metal pair, the nature of the precursors, and their concentrations and relative proportions. Given the large number of parameters involved, a simulation model was developed to investigate the formation of bimetallic nanoparticles in microemulsions prepared *via* the simultaneous reduction of two metal precursors (one-pot method).^25^ This approach enables prediction of the atomic arrangement of the resulting nanoparticles and its correlation with synthesis parameters, allowing precise tuning of the synthesis to obtain predefined structures. The good agreement between experimental and simulated results on Au/Pt nanoparticles validates the model employed in this study.^25,31^

The simulation model describes each metal pair using two key parameters. The first is the chemical reduction rates of each metal precursor, which is related to their standard reduction potentials. Previous experimental work by Troupis *et al.* demonstrated a linear relationship between the logarithm of the rate constant and the redox potential for the reduction of Ag^+^ using different polyoxometalates as reducing agents.^32^ In that study, a linear dependence of log *k*, where *k* is the rate constant of Ag^+^ reduction, on the redox potential was obtained. This behaviour is consistent with the Butler–Volmer relationship, which relates the rate of electron transfer to the electrochemical potential.^33^ In the model, reduction rates are expressed as the percentage of reactants within the colliding micelles that undergo reaction per collision. For Au, whose reduction is instantaneous, *v*_Au_ = 100% is used as reference. Previous studies on the synthesis of Au/Pt nanoparticles showed good agreement between experimental and simulation data when the reduction of Au and Pt were modelled with *v*_Au_ = 100% (*ε*°(AuCl_4_^2−^/Au) = 1.00 V) and *v*_Pt_ = 10% (*ε*°(PtCl_6_^2−^/Pt) = 0.742 V).^25^ Following the experimentally established linear relationship between the logarithm of the reduction rate and the redox potential,^32,33^ the empirical expression: log *v*_A_ = *a* + *bε*°, was used to relate the reduction rate to the standard reduction potential. The constants *a* and *b* were obtained from the Au and Pt reference values. The resulting relationship was then used to estimate the reduction rate of the Pd precursor. For Pd (*ε*°(PdCl_4_^2−^/Pd) = 0.646 V)^34^ the corresponding reduction rate is approximated *v*_Pd_ = 3%. Thus, the reduction rates used in the present study are *v*_Pt_ = 10% and *v*_Pd_ = 3%, meaning that 10% of Pt precursors and 3% of Pd precursors undergo reduction per collision. Consequently, Pt atoms appear earlier than Pd atoms, favouring the development of a Pt-enriched core, while the slower reduction of Pd precursors supplies material for subsequent shell formation.

The second parameter used to characterize a metal pair is the nucleation rate. In contrast to the reduction kinetics, previous studies have proposed that Pd nucleates faster than Pt.^27^ During the early stages of the synthesis, once chemical reduction has occurred, the metal with the higher nucleation rate generates most of the initial nuclei. Subsequent growth of these seeds leads to the formation of the core, onto which the metal with the slower nucleation rate is deposited. This approach, based on differences in nucleation kinetics, has been proposed to explain metal segregation in several bimetallic systems, including Ir/Pd,^35^ Au/Ag,^36^ Au–Pd^37^ or Pt–Pd.^27^ Consistent with this interpretation, computational studies have shown that variations in nucleation rates lead to distinct nanostructure.^38,39^ In particular, the structure of Pt–Pd nanoparticles has been explained by assuming a significantly higher nucleation rate of Pd than for Pt.^27^ To assess whether differences in nucleation kinetics can account for the metal segregation observed in Pt–Pd nanoparticles, nucleation in microemulsions must be reconsidered by including the material intermicellar exchange. The formation of stable nuclei requires the accumulation of a sufficient number of reduced metal atoms within a single micelle. Likewise, subsequent particle growth relies on the continued supply of atoms through the exchange mechanism. Accordingly, the simulation model used in this study combines microemulsion dynamics with classical nucleation theory to describe nanoparticle formation.

According to classical nucleation theory (CNT), nuclei larger than a critical size decrease their free energy upon growth, making further growth thermodynamically favorable. The critical nucleus size is therefore the smallest cluster that can sustain particle growth.^40^ Although CNT is widely applied to nucleation in microemulsions, its validity under nanoconfinement remains uncertain because the small number of atoms challenges the assumptions of continuum thermodynamics.^41^ Nevertheless, CNT remains the most widely used framework for describing nucleation in confined media. Experimental^42^ and computational^43^ studies consistently indicate that critical nucleus size is substantially smaller in microemulsions than in bulk media. This conclusion was already anticipated by Towey *et al.*,^44^ who argued that the formation of 1–2 nm particles necessarily implies nucleation at even smaller sizes. Representative values reported in the literature range from n* = 2 atoms for Au^45,46^ and CdS^47^ to n* = 10 for PtBi^48^ and BaSO_4_,^49^ with intermediate values reported for several inorganic systems.^50–54^

To estimate the critical nucleus size for Pt and Pd, theoretical studies on the stability of small clusters were considered. For Pt, both atomistic and DFT calculations identify Pt_10_ cluster as a particularly stable magic cluster, with the lowest energy structure^55,56^ and exhibiting exceptional stability relative to neighboring sizes.^57^ For Pd, Pd_4_ and Pd_6_ have been reported as the most stable clusters,^58^ although smaller clusters such as Pd_2_ an Pd_3_ may also be stable when supported on surfaces. Based on these findings, 
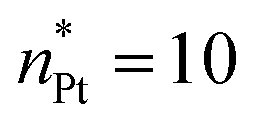
 and three plausible values for Pd 
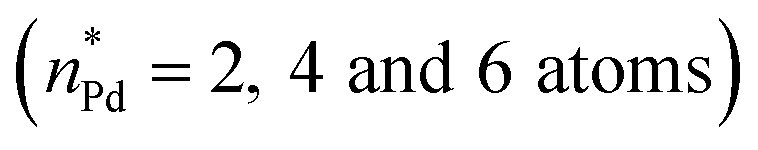
 are considered in this work for homoatomic nucleation. These values are consistent with the lower surface energy of Pd relative to Pt, which is expected to favor a smaller critical nucleus size. This trend is further supported by the lower heat of sublimation of Pd, reflecting its lower cohesive energy relative to Pt. Accordingly, theoretical analyses relating surface energy to crystal nucleation predict a larger critical nucleus size for Pt than for Pd.^58^ This conclusion is also consistent with previous proposals that Pd nucleates significantly faster than Pt.^27^

Regarding the size of the heteroatomic critical nucleus, we considered an effective critical nucleus size, 
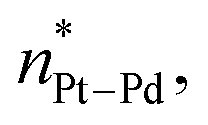
 intermediate between those of the corresponding homoatomic systems. This assumption is consistent with previous theoretical studies in which heteroatomic interactions in bimetallic systems forming solid solutions were represented by intermediate parameters between the corresponding homoatomic interactions.^16–59^ For the Pt–Pd system, Paz-Borbón *et al.*^16^ described the heteroatomic interaction parameters using a weighted average of the Pt–Pt and Pd–Pd interactions, showing that average or slightly Pd-biased values can reproduce the structural and chemical ordering of Pt–Pd nanoalloys. Accordingly, for the case 
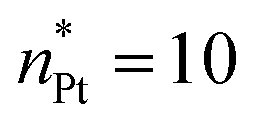
 and 
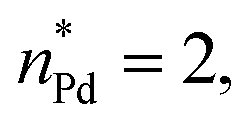
 the heteroatomic critical nucleus size was estimated as the average of the two homoatomic values, yielding 
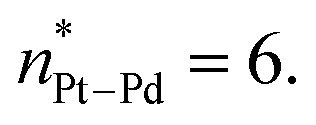
 Applying the same criterion to 
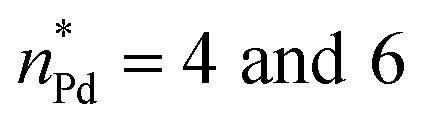
 gives 
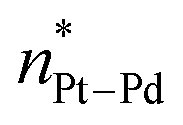
 values of 7 and 8, respectively. Consequently, three representative parameter sets were considered in the simulations: 
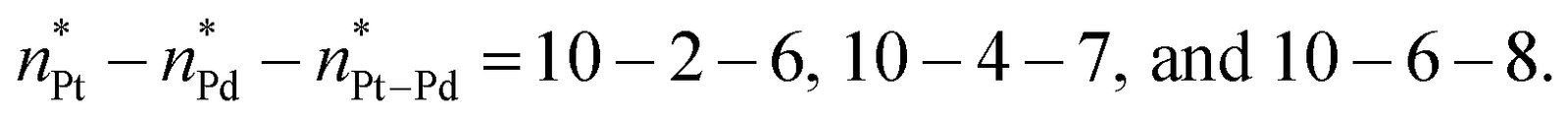
 These nucleation parameters were combined with the higher reduction rate for Pt to investigate the interplay between nucleation and reduction kinetics in the Pt–Pd system. Specifically, the smaller critical nucleus size associated with Pd promotes faster nucleation kinetics, whereas the higher reduction rate of Pt results in its earlier availability for atomic deposition. As a result, nucleation- and reduction-driven effects act in opposite directions, giving rise to competing pathways for metal segregation.

This work aims to elucidate how kinetic parameters, including nucleation kinetics, reactant concentration, and the intermicellar exchange rate, govern metal segregation in Pt–Pd nanoparticles synthesized in microemulsions. The proposed model explains why core–shell structures, homogeneous alloys, or partially segregated structures form under different synthesis conditions and quantifies how the intermicellar exchange rate, the reduction rates of Pt and Pd, and the reactant concentration influence the extent of metal segregation in the final nanoparticles. By identifying the key factors controlling surface composition, this study provides a framework for understanding and tailoring the nanoarchitecture of Pt–Pd nanoparticles, thereby supporting the rational design of improved catalytic materials.

## 2 Computational model

The model reproduces the kinetic course of nanoparticle synthesis within the reverse micelles constituting the microemulsion.

### 2.1 Reaction medium: the microemulsion

The microemulsion is modeled as an ensemble of micelles undergoing random motion. Reactants (the metal salts and the reducing agent) are initially distributed among micelles according to a Poisson distribution, as is commonly adopted in previous studies of microemulsion systems.^48,51,53^ This assumption represents the stochastic distribution of reactants among the microscopic micelles under dilute conditions. Varying the initial number of reactants per micelle allows different concentrations to be simulated. In this study, the average concentration of metal salts ranges from 〈*c*_metal salt_〉 = 8 to 64 atoms per micelle, corresponding to 0.05–0.40 M for a micelle radius of 4 nm. The concentrations of both metal precursors are equal, resulting in a 1 : 1 reactant ratio in the feeding solution. To ensure isolation conditions, the reducing agent is introduced in large excess (〈*c*_R_〉 = 10 〈*c*_metal salt_〉). The one-step procedure is simulated by mixing three microemulsions, each containing one reactant (the two metal salts and the reducing agent).

Micelles undergo Brownian motion and frequently collide. Reactant exchange between micelles occurs only when collisions have sufficient energy to induce fusion-redispersion events. Under these conditions, the colliding micelles transiently fuse, forming an intermicellar channel that enables the reactant transfer between them.^60^ Effective collisions are implemented by randomly selecting 10% of the micelles to merge, exchange material, and subsequently separate. One Monte Carlo step (mcs) corresponds to one complete collision–exchange cycle, after which the material distribution of the micelles is updated according to the exchange rules described below.

### 2.2 Intermicellar material exchange

Material exchange between two colliding micelles is controlled by two parameters that account for the permeability of the transient intermicellar channel formed during fusion–redispersion events. The exchange parameter *k*_ex_ defines the maximum number of individual species (metal precursors, reducing agent or isolated metal atoms) that can be transferred during a single collision. Transfer proceeds from the more populated to the less populated micelle, driven by the concentration gradient. The flexibility parameter *f* defines the maximum size of atomic aggregates that can traverse the channel. While isolated species are mainly limited by the contact time between micelles, aggregate transfer is primarily restricted by the channel diameter.

Material exchange depends on the contents of the colliding micelles. Isolated atoms preferentially migrate toward micelles containing growing aggregates, whereas, when both micelles contain particles, the smaller aggregate is transferred to the larger one whenever the channel size permits (*f*). This mechanism reproduces Ostwald ripening through particle transfer between micelles. Exchange continues until the channel size no longer allows aggregate transfer.^61^

The values of *k*_ex_ and *f* reflect the physicochemical properties of the microemulsion. More flexible surfactant films generate larger and longer-lived intermicellar channels, allowing the transfer of both a larger number of individual species (high *k*_ex_) and larger aggregates (high *f*). Consequently, both parameters are closely coupled and increase together. For example, the flexible isooctane/Tergitol/water microemulsion was successfully described using *f* = 30 and *k*_ex_ = 5,^25^ whereas the more rigid AOT/*n*-heptane/water system was modelled with *f* = 5, *k*_ex_ = 1.^34^

### 2.3 Chemical reduction

Chemical reduction occurs when, after material redistribution during collisions, the metal salt and the reducing agent are located within the same micelle. As noted above, reduction rates are defined as the percentage of reactants within the colliding micelles that undergo reaction per collision. These rates are related to the standard reduction potentials *via* the Butler–Volmer equation. In this framework, metal precursors can be characterized by their reduction rates, with higher reduction potentials corresponding to faster reduction kinetics. Based on this relationship, the reduction rates of Pt (*ε*°(PtCl_6_^2−^/Pt) = 0.742 V) and Pd (*ε*°(PdCl_2_/Pd) = 0.646 V)^33^ can be approximated as *v*_Pt_ = 10% and *v*_Pd_ = 3%, respectively. This implies that, provided sufficient reducing agent is available, 10% of Pt precursors within colliding micelles are reduced per collision, whereas only 3% of Pd precursors are reduced per collision.

When both reductions are possible within the same micelle, the reduction order is selected randomly. Metal salts that do not react remain in the micelle and can be reduced in subsequent collisions. As the synthesis progresses, an increasing number of micelles therefore contain both reactants and products simultaneously.

### 2.4 Nucleation

During the synthesis, Pt and Pd atoms accumulate within individual micelles through chemical reduction and intermicellar exchange. Cluster stability is described by introducing a critical nucleus size (n*), defined as the minimum number of atoms required to form a stable nucleus capable of sustained growth. Clusters smaller than n* are assumed to dissociate, and the resulting atoms are redistributed individually among micelles, with exchange governed by *k*_ex_. Once n* is exceeded, a stable nucleus is formed that can continue growing and is transferred between micelles as a single aggregate, with exchange limited by the channel size (*f*).

To account for the different nucleation behavior of Pt, Pd, and mixed Pt–Pd clusters, the model defines three critical nucleus sizes: 
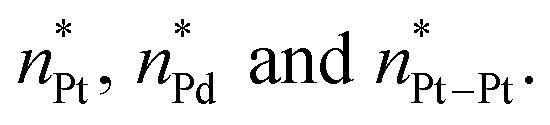
 corresponding to homoatomic Pt, homoatomic Pd, and heteroatomic nucleation, respectively. Thus, 
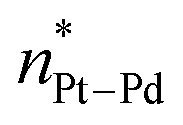
 represents the minimum number of Pt and Pd atoms required to form a stable mixed nucleus, independently of the critical sizes for the corresponding pure metals. Based on the analysis presented above, three parameter sets were considered: 
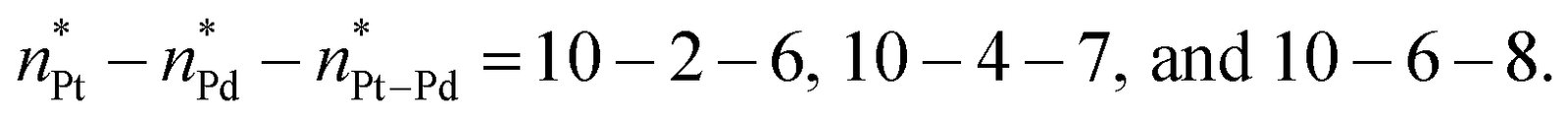


At each Monte Carlo step, the sequence of Pt and Pd atom deposition is recorded for every growing nanoparticle.

### 2.5 Nanoparticle growth

Once stable clusters have formed, nanoparticle grow through intermicellar exchange, which enables new metal atoms to be transferred to nucleated micelles and deposited on the surface of growing aggregate. Growth is governed by three mechanisms, depending on the contents of the colliding micelles.

#### (i) Growth by reactant transfer

When one micelle contains a growing particle and the other contains only reactants, the reactants are preferentially transferred to the nucleated micelle. Chemical reduction then occurs on the particle surface, promoting the growth of existing nanoparticles rather than the formation of new nuclei.

#### (ii) Autocatalytic growth

When both colliding micelles contain growing particles, reactants are preferentially transferred to the micelle containing the larger aggregate, whose greater surface area enhances catalytic activity. To account for this effect, the reduction rate in micelles containing a growing particle is assumed to be twice that in micelles without aggregates. Mechanisms (i) and (ii) therefore reproduce autocatalytic nanoparticle growth through preferential reduction at surface.

#### (iii) Ostwald ripening

When two nucleated micelles collide, the smaller aggregate is transferred to the micelle containing the larger one, provided that the intermicellar channel is sufficiently wide, as determined by the flexibility parameter (*f*). Once both particles are located within the same micelle, the smaller aggregate dissolves and its atoms are incorporated into the larger particle, thereby reducing the overall surface free energy. Aggregate transfer depends exclusively on particle size and is independent of composition. To preserve the metal distribution, the deposition sequence of the transferred aggregate is incorporated into the surface of the larger particle. This mechanism reproduces Ostwald ripening^63,64^ under confinement and can operate throughout the synthesis. The present implementation follows the approach originally proposed^65^ and subsequently adopted in related simulation studies.^48,62^ By contrast, other computational models attribute a greater contribution to particle coagulation.^45,46,61^

### 2.6 Final nanostructure

The input for each simulation run specifies the exchange parameters (*f* and *k*_ex_), the nucleation values 
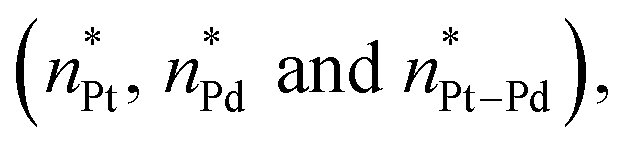
 the reduction rate values (*v*_Pt_ and *v*_Pd_), and the initial reactant concentrations. Under the specific conditions defined by each combination of input values, a nanoparticle can grow within each micelle through the deposition of Pt and Pd atoms as the synthesis progresses. The deposition sequence of Pt and Pd atoms is recorded for each nanoparticle throughout the simulation. Equilibrium is reached when all reactants have been consumed and no further intermicellar exchange occurs. At this stage, one simulation run is considered complete. The results is a population of micelles, each of which can contain a nanoparticle with fully defined deposition sequence of Pt and Pd atoms. Next, assuming a spherical geometry, each nanoparticle is divided into ten concentric layers, preserving the order in which Pt and Pd were deposited. The percentage of Pt is then calculated for each layer of every nanoparticle. Finally, the results are averaged over 1000 simulation runs. The final structure is represented by histograms showing the number of particles with a given percentage of Pt in each layer, from the core to the shell.

### 2.7 Model validation

The simulation model was validated by direct comparison with experimental results reported previously.^25^ Au/Pt particles synthesized using a water/Tergitol/isooctane microemulsion were characterized by HR-STEM (High-Resolution-Scanning Transmission Electron Microscopy) and their radial metal distribution was determined by cross-sectional EDX (Energy Dispersive X-ray spectroscopy). The excellent agreement between the experimental composition profiles and those predicted from the simulated structures demonstrates the predictive capability of the model.

## 3 Results and discussion

Pt–Pd nanoparticle formation was simulated using the reduction rates and critical nucleus sizes defined in the previous section. The reduction rates were fixed at *v*_Pt_ = 10% and *v*_Pd_ = 3%, while the critical nucleus size of Pt was set to 
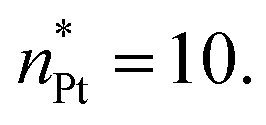
 To evaluate the influence of Pd nucleation kinetics, three values of the Pd critical nucleus size were considered 
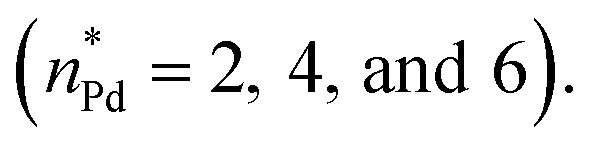
 The corresponding heteroatomic critical nucleus size were taken as intermediate between those of the two pure metals, giving 
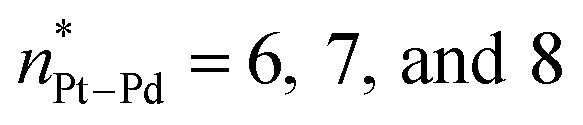
 for 
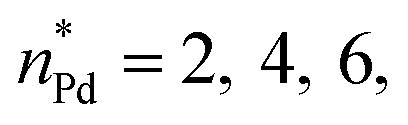
 respectively. Accordingly, the simulations were performed using the parameter setse: 
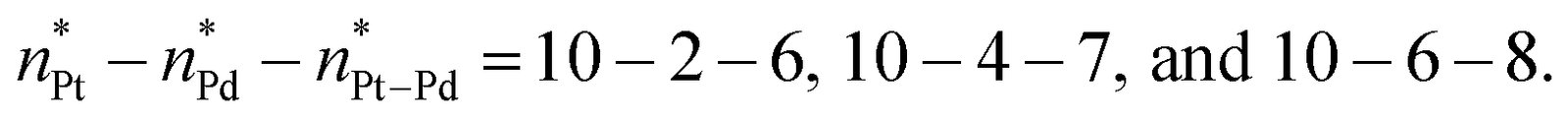


### 3.1 Structural predictions as a function of critical nucleus sizes

To isolate the effect of the critical nucleus size on the final nanoparticle structure, Fig. 1A shows the simulated metal distribution in the absence of a nucleation barrier, assuming that any two-atom cluster is stable regardless of composition 
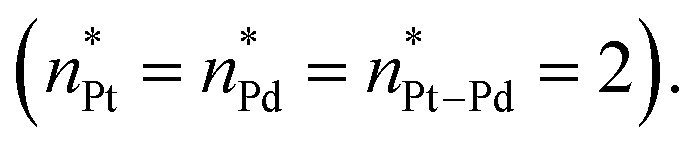
 Under these conditions, the nanoparticles exhibit a nearly core–shell structure, with a Pt-rich core, compositionally mixed intermediate layers, and a Pd-enriched surface. This distribution is a direct consequence of the different reduction kinetics of Pt and Pd. Because Pt precursors are reduced more rapidly, Pt atoms are preferentially incorporated during the early stages of growth, giving rise to a Pt-rich core. As the reaction proceeds, Pd reduction progressively contributes to particle growth, producing mixed intermediate layers. Once the Pt precursor is depleted, subsequent growth is dominated by Pd deposition, leading to Pd enrichment in the outermost layers. This limiting case provides a useful reference for assessing the structural changes induced by finite critical nucleus sizes.

**Fig. 1:**
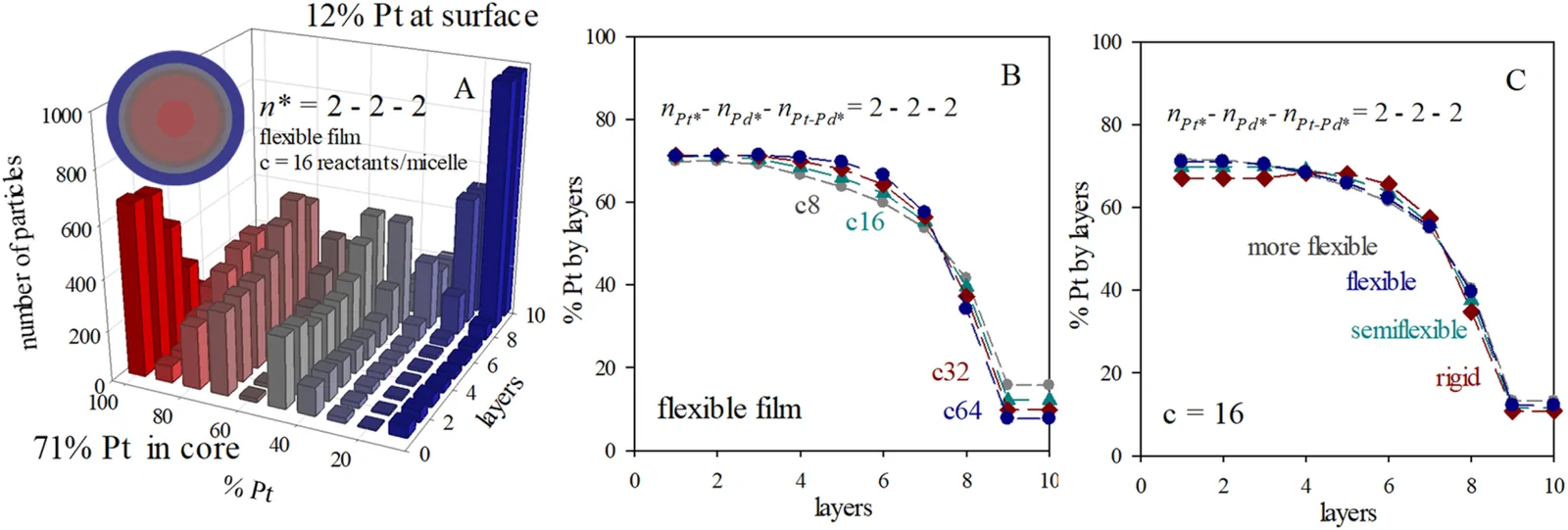
(A) Number of particles as a function of Pt content (%) from the core to the surface (layer by layer) in the absence of a nucleation barrier 
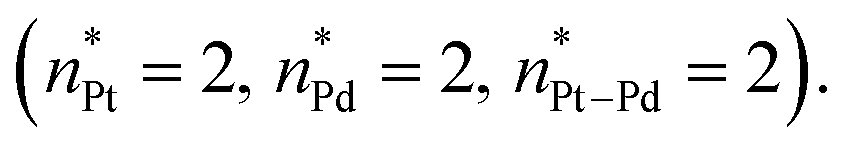
 Color code: blue (0–45% of Pt), grey (45–55% of Pt), red (55–100% of Pt), with lower red intensity indicating lower Pt content. Circles represent the nanoparticle structure in concentric layers, using the same color scheme. Simulation parameters: average reactant concentration 〈*c*〉 = 16 reactants per micelle; 〈*c*_reductor_〉 = 10 〈*c*〉, 1 : 1 reactants ratio in the feed solution; flexible film (*f* = 30, *k*_ex_ = 5). (B) Average Pt content (%) as a function of layer number (1 = innermost, 10 = outermost) in the absence of a nucleation barrier 
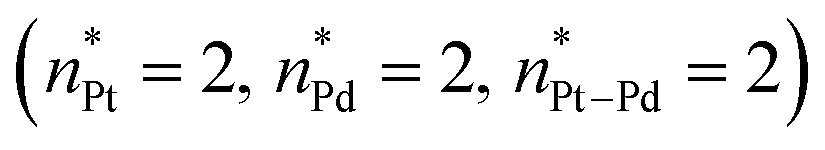
 for different initial average concentration: 〈*c*〉 = 8, 16, 32, and 64 reactants per micelle (grey, cyan, red and blue, respectively). Simulation parameters: 〈*c*_reductor_〉 = 10 〈*c*〉; flexible film (*k*_ex_ = 5, *f* = 30). (C) Average Pt content% as a function of layer number for different film flexibilities: rigid (*f* = 5, *k*_ex_ = 1, red), semiflexible (*f* = 15, *k*_ex_ = 3, cyan), flexible (*f* = 30, *k*_ex_ = 5, blue) and more flexible film (*f* = 40, *k*_ex_ = 8, grey). Simulation parameters: reduction rates: *v*_Pt_ = 10%, *v*_Pd_ = 3% reaction per collision.

For comparison purposes, Fig. 1B shows the compositional profiles obtained at different reactant concentrations in the absence of nucleation constraints. Under these conditions, reactant concentration has only a minor influence on metal segregation because criterion is readily satisfied regardless of concentration. As a result, the final compositional is governed primarily by the difference in the reduction kinetics of Pt and Pd rather than by nucleation. The same qualitative behavior is observed for different surfactant film flexibilities (Fig. 1C), indicating that neither reactant concentration nor intermicellar exchange significantly affects metal segregation when the nucleation barrier is negligible.

Fig. 2 shows the predicted nanoparticle structures for the three proposed sets of critical nucleus sizes, while all other variables are kept constant. Although Pt is reduced more rapidly than Pd, its larger critical nucleus size 
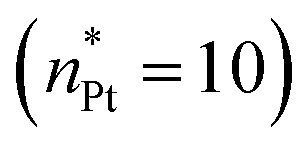
 delays the formation of stable Pt nuclei. Consequently, the nanoparticle core is significantly more mixed than in the limiting case shown in Fig. 1, where 
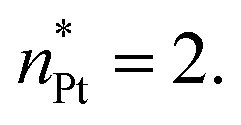
 The delayed nucleation of Pt allows Pd atoms, despite their slower reduction, to participate in the early stages of particle growth. In addition, the smaller critical nucleus size for heteroatomic nucleation 
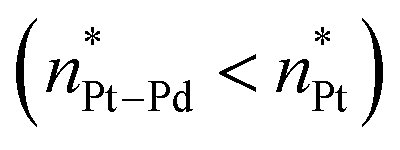
 facilitates the formation of mixed Pt–Pd nuclei. Together, the faster nucleation of Pd 
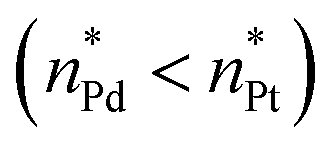
 and the preferential formation of mixed nuclei 
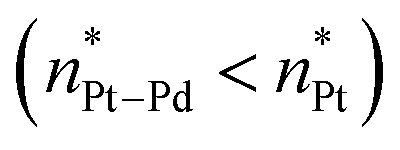
 promote extensive mixing within the inner nanoparticle layers.

**Fig. 2:**
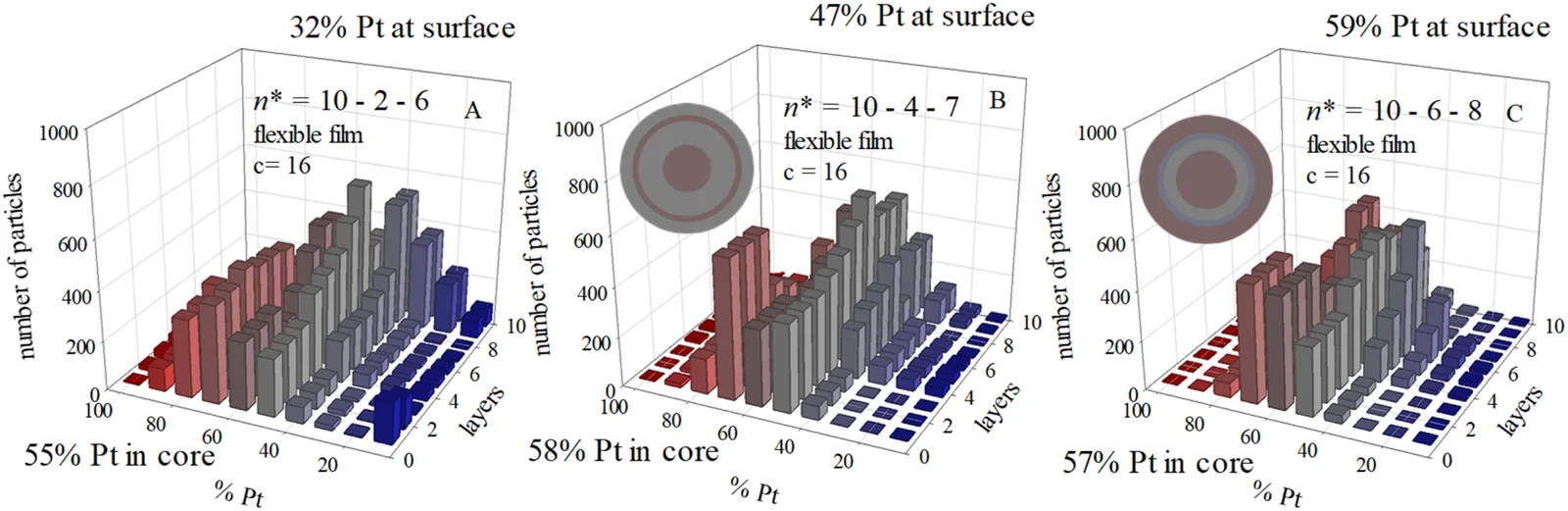
Number of particles as a function of the Pt content (%) from the core to the surface (layer by layer) for the proposed sets of critical nucleus sizes. Color code: blue (0–45% of Pt), grey (45–55% of Pt), red (55–100% of Pt), with lower red intensity indicating lower Pt content. Circles in each histogram represent nanoparticle structure in concentric layers using the same color scheme. Simulation parameters: reduction rates *v*_Pt_ = 10%, *v*_Pd_ = 3% reaction per collision; flexible surfactant film (*k*_ex_ = 5, *f* = 30); average reactant concentration 〈*c*〉 = 16 reactants per micelle; 〈*c*_reductor_〉 = 10 〈*c*〉; critical nucleus size sets: (A) 
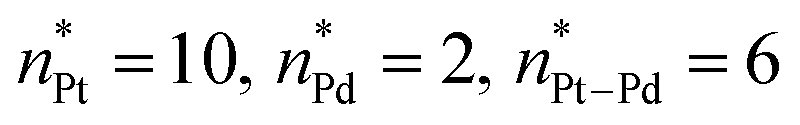
 (n* = 10–2–6); (B) 
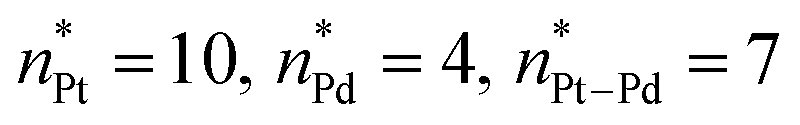
 (n* = 10–4–7); (C) 
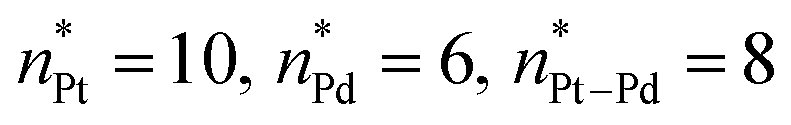
 (n* = 10–6–8).

Regarding the surface composition, a progressive Pt enrichment is observed as both 
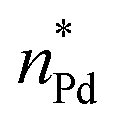
 and 
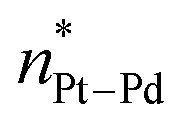
 increase (from left to right in Fig. 2). This trend indicates that higher critical nucleus sizes for Pd and Pt–Pd heteronucleation favor Pt incorporation at the nanoparticle surface. To identify which nucleation parameter primarily drives this Pt surface enrichment, the structures were analysed by varying each parameter independently. Although this approach leads to unrealistic combinations of critical nucleus sizes, it provides a useful means of isolating their individual contributions. For a more direct comparison of the resulting structures, Fig. 3 presents the average Pt content (%) as a function of layer number. Varying the heteroatomic critical nucleus size 
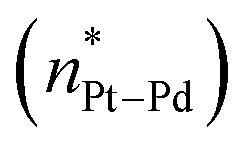
 produces a pronounced change in metal segregation (Fig. 3A). In contrast, when the heteroatomic nucleation parameter is kept constant, variations in the critical nucleus size of pure Pd 
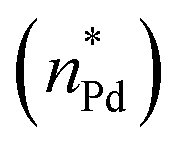
 have only a negligible effect on the compositional profile (Fig. 3B). These results indicate that 
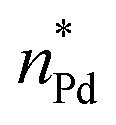
 plays only a minor role in determining the final structure. A plausible explanation is that the relatively large critical nucleus size of Pt leads to the accumulation of a substantial concentration of free Pt atoms in the reaction medium before Pd atoms begin to appear. Under these conditions, Pd nucleation is expected to proceed predominantly through heteroatomic co-nucleation with Pt 
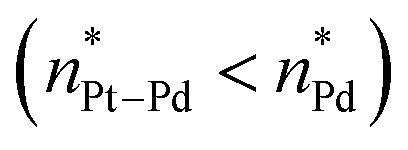
 rather than through the formation of pure Pd nuclei. This behaviour is reflected in the compositional profiles, which evolve from a core–shell-like structure (Pt-rich core and Pt-depleted surface) when conucleation is favoured (
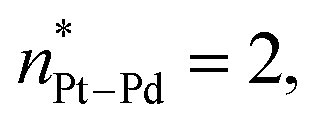
Fig. 3A) to a highly intermixed structure across all layers when conucleation is hindered (
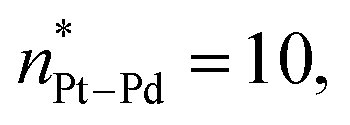
Fig. 3A). This behaviour has a direct impact on the surface composition. Because the nucleation barrier for pure Pt is high, increasing the heteronucleation critical nucleus reduces Pt incorporation during the early stages of nanoparticle growth. As a result, more Pt remains available in the reaction medium until later stages of the synthesis, promoting its deposition at or near the particle surface. In summary, increasing the critical nucleus size for heteronucleation delays Pt incorporation, leading to more homogeneous elemental mixing throughout the nanoparticle while enhancing Pt enrichment at the surface.

**Fig. 3:**
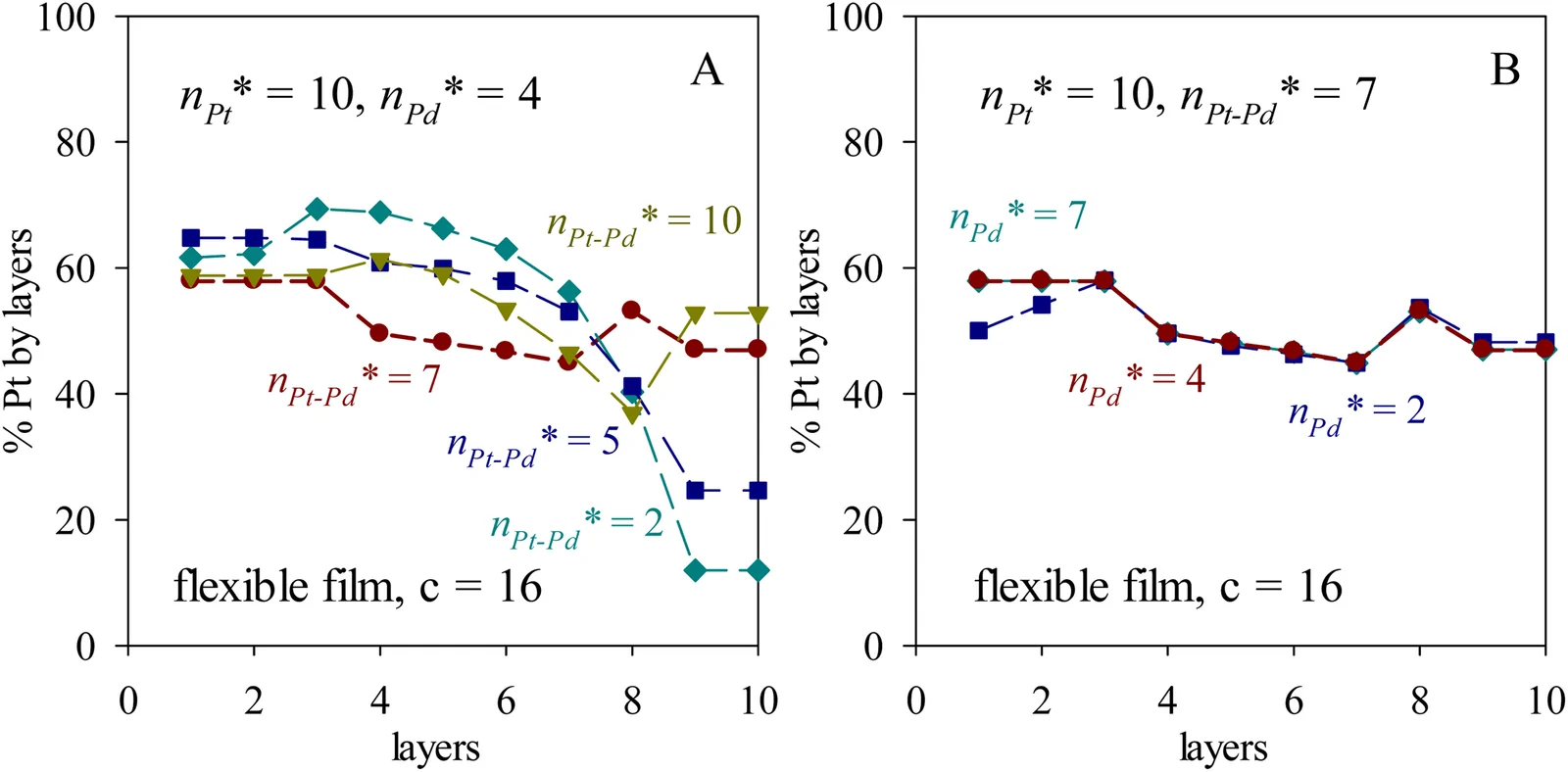
Average Pt content (%) as a function of layer number (1 = innermost, 10 = outermost). (A) The critical nucleus sizes for homoatomic nuclei are kept constant 
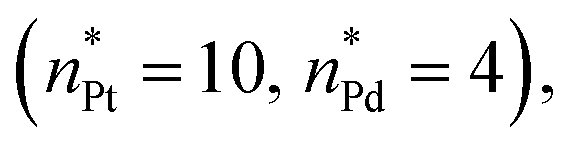
 while the critical nucleus size for heteroatomic nucleation 
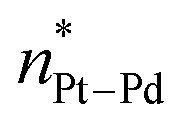
 is varied (
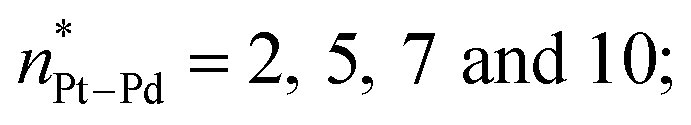
 cyan, blue, red and green, respectively). (B) The critical sizes for Pt homoatomic nucleation and Pt–Pd heteronucleation are kept constant 
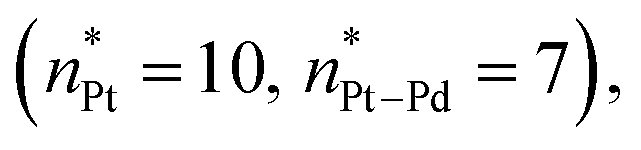
 while the critical nucleus size for Pd homoatomic nucleation 
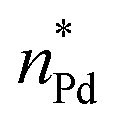
 is varied (
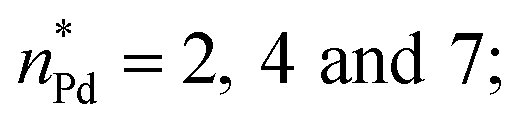
 blue, red and cyan, respectively). Simulation parameters: reduction rates *v*_Pt_ = 10%, *v*_Pd_ = 3% reaction per collision; surfactant film flexibility (*k*_ex_ = 5, *f* = 30); average reactant concentration 〈*c*〉 = 16 reactants per micelle; 〈*c*_reductor_〉 = 10 〈*c*〉.

### 3.2 Structural predictions as a function of reactants concentration

In contrast, metal segregation is strongly affected by reactant concentration for all proposed sets of critical nucleus sizes, as shown in Fig. 4. The lowest concentration that could be analyzed is 〈*c*〉 = 16 reactants per micelle, since lower values do not lead to nanoparticle formation, or produce only negligible amounts of nanoparticles, because the nucleation requirements cannot be readily satisfied. In all cases, increasing the reactant concentration results in a more pronounced compositional depth profile, *i.e.*, better segregation of the two metals. This is because higher concentrations facilitate fulfilment of the nucleation requirements, allowing Pt to be incorporated during the early stages of nanoparticle growth despite the large values of 
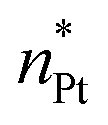
 and 
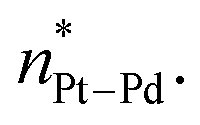
 Consequently, Pt is depleted more rapidly from the reaction medium, leaving less Pt available for deposition during the later stage of growth. As a result, the compositional profiles become progressively deeper as the reactant concentration increases. This trend is clearly reflected in the final nanostructure structures shown in Fig. 4, where the surface becomes progressively richer in Pd (brighter blue) with increasing reactant concentration for all three proposed sets of critical nucleus sizes.

**Fig. 4:**
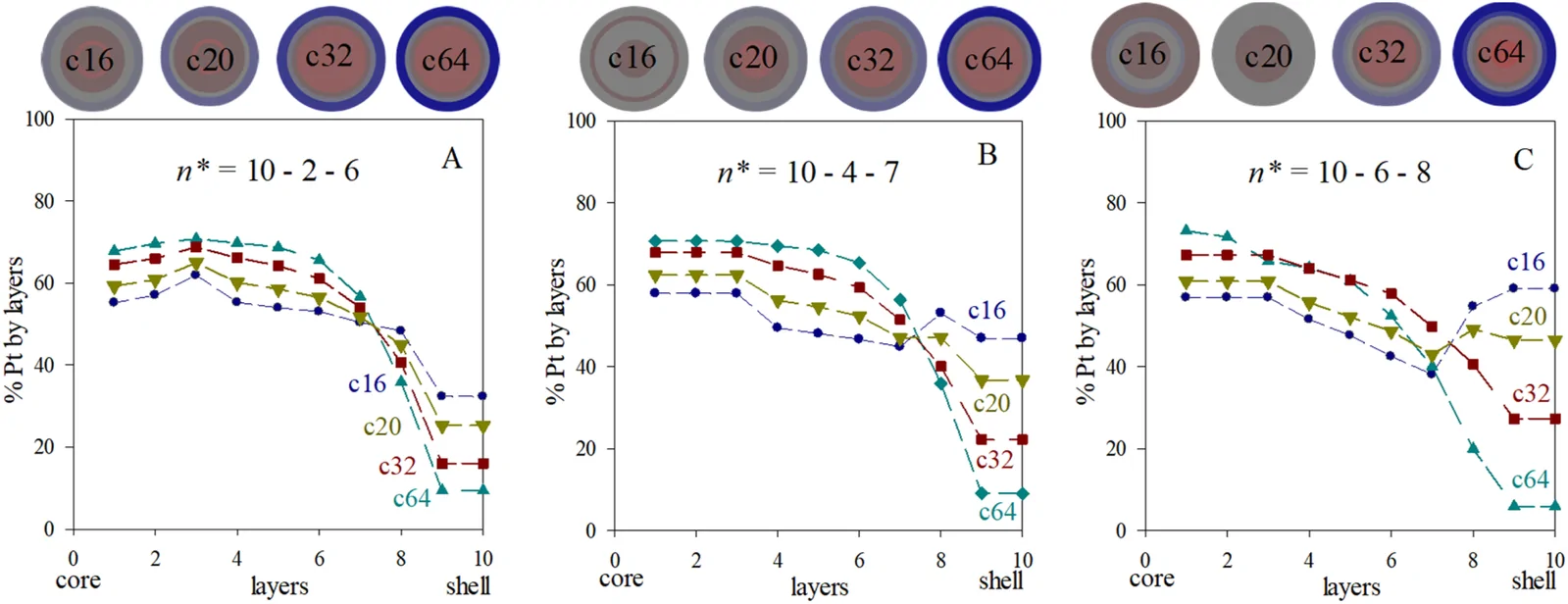
Average Pt content (%) as a function of layer number for different average reactant concentrations. 〈*c*〉 = 16, 20, 32, 64 reactants per micelle are represented in blue, green, red and cyan, respectively. (A) 
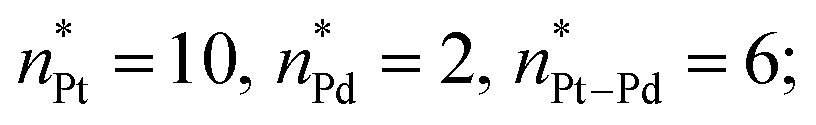
 (B) 
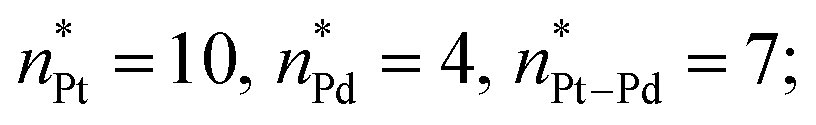
 (C) 
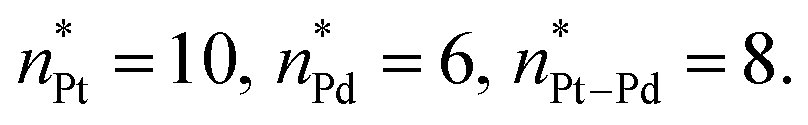
 Simulation parameters: reduction rates of *v*_Pt_ = 10% and *v*_Pd_ = 3% per collision; 〈*c*_reductor_〉 = 10 〈*c*〉; flexible surfactant film (*f* = 30, *k*_ex_ = 5). Circles represent the nanoparticle structure in concentric layers using the following color scheme: blue (0–45% of Pt), grey (45–55% of Pt), red (55–100% of Pt). Brighter blue indicates a higher Pd content.

Focusing on the low-concentration profiles, it can be seen that, as the critical nucleus sizes increase, the metal segregation evolves from a quasi-core–shell structure with a Pt-enriched core and a Pd-enriched shell (see blue curve in Fig. 4A) to a fully mixed structure (see Fig. 4B), and finally to a structure with Pt-enriched surface (see Fig. 4C). It should also be noted that, when low reactant concentrations are combined with large critical nucleus sizes, atypical segregation profiles are obtained, in which the intermediate layers contain less Pt than the outermost layer. These results are discussed further in the Section 3.4.

### 3.3 Structural predictions as a function of microemulsion composition

Fig. 5 shows the compositional profiles obtained for the proposed sets of critical nucleus sizes at different surfactant film flexibilities, and two reactant concentrations. The first point to note is that, in all cases, increasing film flexibility leads to a higher Pt content at the nanoparticle surface. In contrast, the effect of film flexibility is negligible in the core, regardless of reactant concentration. In other words, changes in microemulsion composition have little influence on the early stages of nanoparticle formation but a pronounced effect on the later stages of nanoparticle growth.

**Fig. 5:**
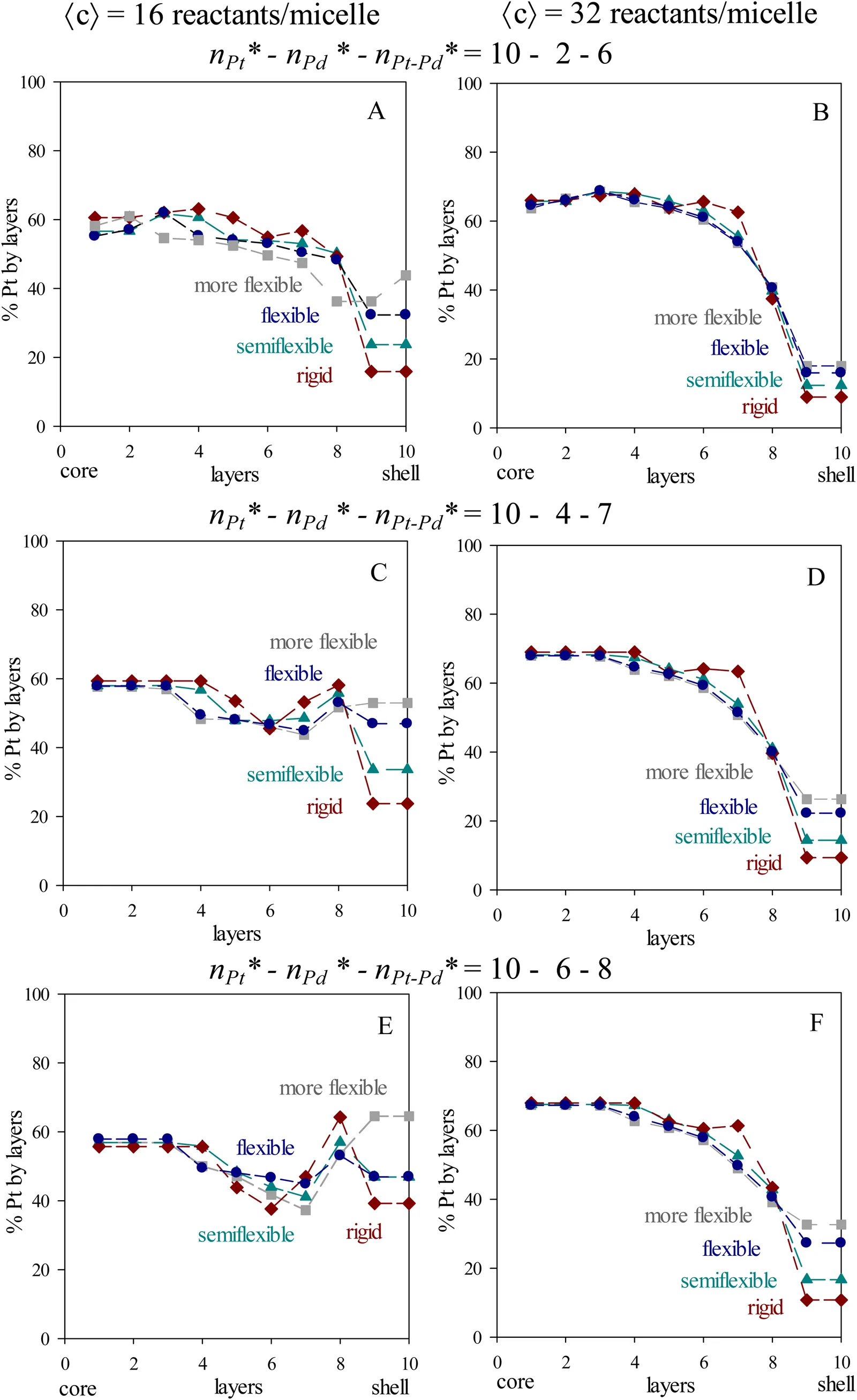
Average Pt content (%) as a function of layer number for different film flexibilities and two average reactant concentrations. Panels (A) and (B): 
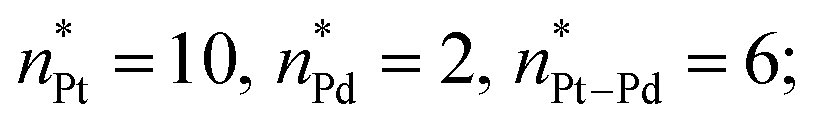
 panels (C) and (D): 
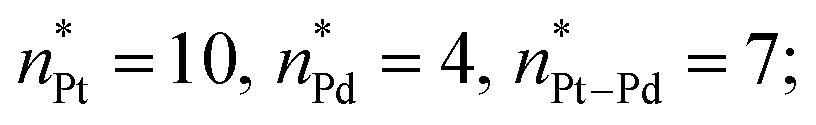
 panels (E) and (F): 
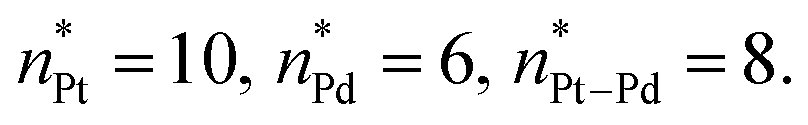
 Left column: 〈*c*〉 = 16 reactants per micelle. Right column: 〈*c*〉 = 32 reactants per micelle. Film flexibility: rigid (*f* = 5, *k*_ex_ = 1, red), semiflexible (*f* = 15, *k*_ex_ = 3, cyan), flexible (*f* = 30, *k*_ex_ = 5, blue) and more flexible film (*f* = 40, *k*_ex_ = 8, grey). Simulation parameters: reduction rates: *v*_Pt_ = 10%, *v*_Pd_ = 3% reaction per collision; 〈*c*_reductor_〉 = 10 〈*c*〉.

Second, high reactant concentrations consistently lead to core–shell-type structures, in which the averaged Pt content decreases monotonically from the core to the surface, regardless of film flexibility (see right column). This behaviour is also observed at low reactant concentration, but only when the critical nucleus sizes are small (see red curve in Fig. 5A, n* = 10–2–6). In contrast, the combination of low reactant concentration and large critical nucleus sizes gives rise to anomalous structures, in which intermediate layers may contain more Pt than the outermost layer.

To summarize, Fig. 6 focuses on surface composition, the key parameter from a catalytic perspective. The surface Pt content is plotted as a function of the initial average reactant concentration for the three proposed sets of critical nucleus sizes and the no-nucleation-barrier case, considering two surfactant film flexibilities. Three main factors are found to influence nanoparticle surface composition. First, the critical nucleus sizes have a strong effect, particularly at low reactant concentrations. Higher reactant concentrations accelerate reduction, increasing the availability of metal atoms for nucleation and reducing the influence of the critical nucleus sizes. Second, higher reactant concentrations consistently reduce the Pt content at the nanoparticle surface by promoting earlier Pt incorporation into the growing particles, leaving less Pt available for deposition during the final stages of growth. Third, increasing surfactant film flexibility leads to a higher Pt content at the surface and broadens the concentration range over which surface composition is affected by critical nucleus sizes. This behaviour can be explained as follows. On the one hand, more flexible films give rise to fast-kinetic nanoreactors because they provide faster intermicellar exchange of reactants, accelerating both reduction and nucleation and promoting the incorporation of more Pt into the inner and intermediate layers. However, this trend is opposite to that observed in the simulations: higher film flexibility should lead to a lower Pt content at the surface. On the other hand, increased flexibility also facilitates the intermicellar exchange of larger particles, thereby promoting Ostwald ripening. In this process, larger particles grow through the deposition of smaller ones. This mechanism enables the Pt incorporated during the early stages of the formation of the smaller particles to be transferred to the larger particles, where it ultimately becomes part of their outer layers of the larger particle. Consequently, a greater contribution of Ostwald ripening results in enhanced Pt enrichment at the nanoparticle surface. Fig. 7A illustrates this phenomenon. Consider a collision between a micelle containing a large particle composed of both Pd and Pt and another micelle containing a small particle composed predominantly of Pt atoms reduced during the early stages of the synthesis. A small intermicellar channel size (*f* = 1) does not allow the small particle to be transferred to the micelle containing the large particle, thereby preventing Ostwald ripening. In contrast, ripening becomes possible when the intermicellar channel is sufficiently large (*f* = 3) to allow the small particle to be transferred, and the Pt atoms are subsequently deposited onto the surface of the larger particle, resulting in Pt enrichment at the nanoparticle surface.

**Fig. 6:**
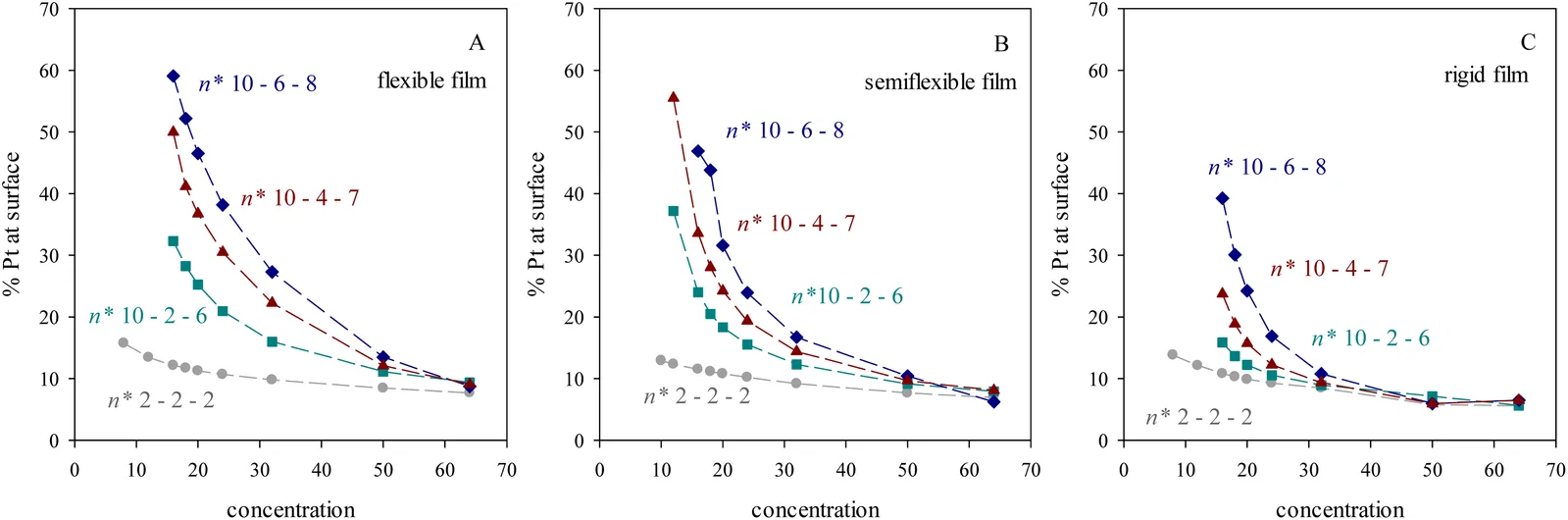
Average Pt content (%) at the nanoparticle surface as a function of the initial average reactant concentration. (A) *f* = 30, *k*_ex_ = 5; (B) *f* = 15, *k*_ex_ = 3; (C) *f* = 5, *k*_ex_ = 1. Critical nucleus size sets: n* 2–2–2 (grey), n* 10–2–6 (cyan), n* 10–4–7 (red), n* 10–6–8 (blue). Simulation parameters: reduction rates *v*_Pt_ = 10%, *v*_Pd_ = 3% reaction per collision, 〈*c*_reductor_〉 = 10 〈*c*〉.

**Fig. 7:**
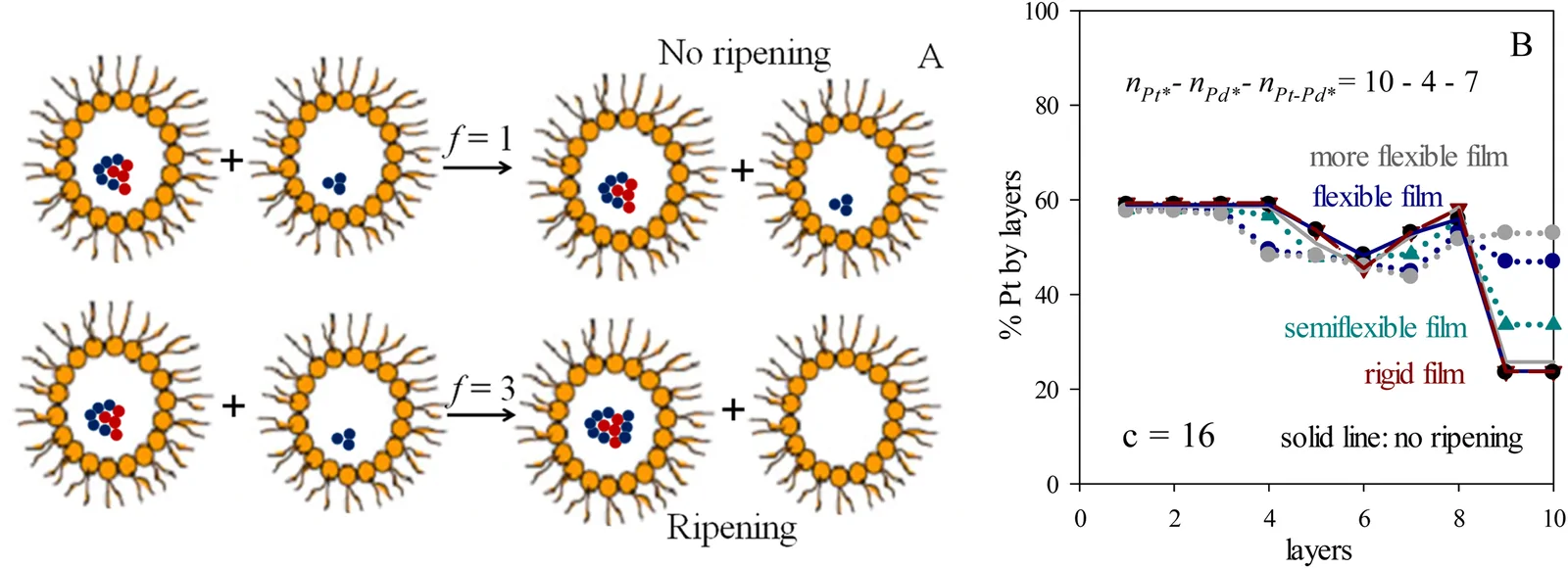
(A) Scheme illustrating the Pt enrichment at the nanoparticle surface due to Ostwald ripening (Pt and Pd are represented in blue and red, respectively). (B) Average Pt content (%) as a function of layer number (1 = innermost layer, 10 = outermost layer) for the critical nucleus sizes set 
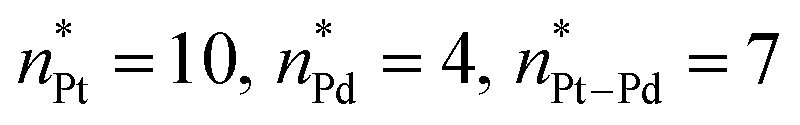
 and different surfactant film flexibilities: rigid (*k*_ex_ = 1, *f* = 5, red), semiflexible (*k*_ex_ = 3, *f* = 15, cyan), flexible (*k*_ex_ = 5, *f* = 30, blue) and more flexible (*k*_ex_ = 8, *f* = 40, grey), shown as symbols and dotted lines. Solid lines show the corresponding results in the absence of ripening (*f* = 1), using the same colour scheme. Simulation parameters: reduction rates of *v*_Pt_ = 10% and *v*_Pd_ = 3% reaction per collision; initial average reactant concentration 〈*c*〉 = 16 reactants per micelle; 〈*c*_reductor_〉 = 10 〈*c*〉.

To test the hypothesis that ripening is responsible for the enrichment of Pt at the surface when more flexible films are used, Fig. 7B shows compositional profiles for the studied flexibility values (dotted lines) together with those obtained in absence of ripening (solid lines) for comparison. In the latter case, film flexibility is defined solely by the intermicellar exchange constant, *k*_ex_, since the channel size is kept small and constant (*f* = 1). Although this provides only a simplified description of interfacial flexibility, it allows the effect of ripening to be isolated. All curves overlap when ripening is suppressed (solid lines), confirming that the contribution of ripening to particle growth is responsible for Pt enrichment at the nanoparticle surface.

### 3.4 Kinetic study

In most cases, the influence of the different parameters (critical nucleus sizes, reactant concentration and surfactant film flexibility) on the final nanostructure has been satisfactorily explained. We now focus on the atypical compositional profiles in which the Pt content does not decrease monotonically from the core to the surface (see Fig. 5C and E). These profiles are observed at low concentrations when large critical nucleus sizes are considered. To understand the origin of these structures, Fig. 8 shows the number of Pt atoms (red lines) and Pd atoms (blue lines) present in the reaction medium as the synthesis progresses for the three proposed critical nucleus size sets, using two different microemulsions while maintaining a low reactant concentration. The dotted lines represent the total numbers of Pt and Pd atoms in the reaction medium as the reduction proceeds, clearly showing the slower reduction of Pd. The higher intermicellar exchange rate associated with the flexible film promotes more frequent encounters between the metal precursors and the reducing agent, thereby accelerating the overall reduction process at the beginning of the synthesis. The resulting metal atoms can either remain free in the reaction medium or become incorporated into growing nanoparticles. Consequently, the initial increase in the number of reduced atoms is slower for the rigid film than for the flexible film. This difference is reflected in the slopes of the curves representing the total number of reduced atoms (dotted lines) and the number of free atoms (solid lines). Overall, the initial slopes are less steep for the rigid film (left column) than for the flexible film (right column). Since the reduction rates are independent of the critical nucleus sizes, they remain unchanged across all panels within each column. Once produced, the metal atoms remain free in the reaction medium (solid lines) until they are incorporated into a nanoparticle (dashed-dotted lines), after the nucleation requirements have been satisfied.

**Fig. 8:**
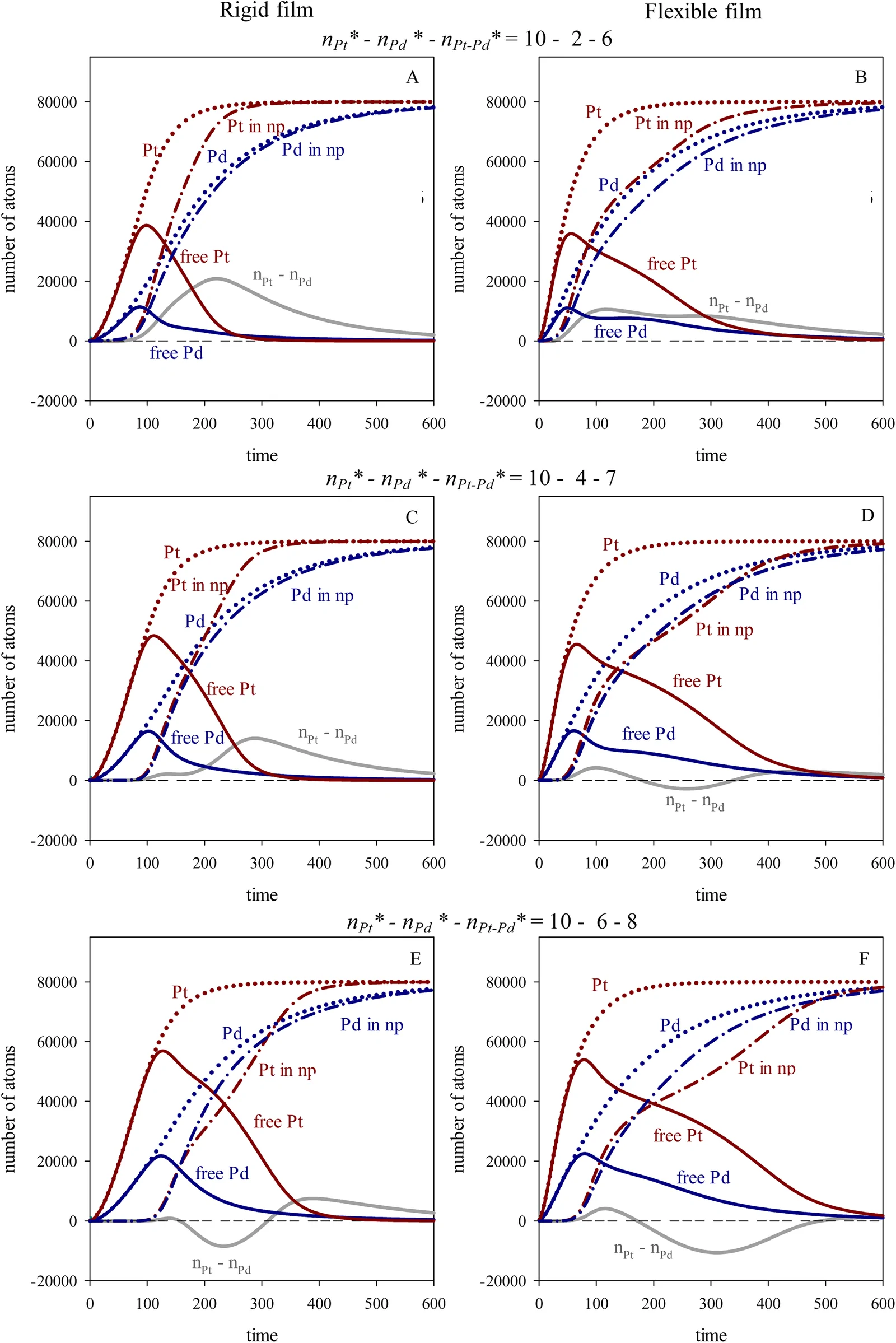
Total number of atoms (dotted), free atoms (solid) and atoms incorporated into the nanoparticles (dashed-dotted) as a function of time. Pt is shown in red, and Pd in blue. Grey lines represent the difference between the number of Pt and Pd atoms incorporated into the nanoparticles (free atoms are not included). Panels (A) and (B): n* 10–2–6, panels (C) and (D): n* 10–4–7, panels (E) and (F): 10–6–8. Left column: rigid film. Right column: flexible film. Simulation parameters: reduction rates of *v*_Pt_ = 10% and *v*_Pd_ = 3%; 〈*c*〉 = 16 atom per micelle, 〈*c*_reductor_〉 = 10 〈*c*〉.

The number of free atoms reaches a maximum, after which it decreases as the atoms are incorporated into nanoparticles. In all cases, the number of free Pt atoms is much higher than that of free Pd atoms because Pt nucleation is more difficult. Comparing the panels from top to bottom (*i.e.*, as the critical nucleus sizes increase), the maxima become higher as the nucleation requirements become harder, as expected. Comparing the left and right columns, however, reveals an unexpected trend: the greater the film flexibility, the longer the atoms remain free in the reaction medium. This behavior can be explained as follows. Free Pt and Pd atoms are removed from the reaction medium when they satisfy the nucleation requirements within a micelle and they cluster. This process is limited by *k*_ex_, which restricts the number of atoms that can be exchanged during each intermicellar collision. In addition, when a micelle containing free atoms collides with another containing a nanoparticle, the free atoms can be transferred to the nucleated micelle and incorporated into the growing particle. This process is also limited by *k*_ex_. Therefore, increasing *k*_ex_ would be expected to accelerate the consumption of free atoms, leading to a steeper decrease in the curves after the maximum, contrary to the observed trend. To explain this behaviour, it should be noted that greater film flexibility promotes a larger contribution of ripening to particle growth, concentrating the material into a smaller number of micelles. After a ripening event, one micelle contains a larger nanoparticle, whereas the other becomes empty. This effect is reflected in the smaller number of micelles containing a particle as film flexibility increases shown in Fig. 9A. As the number of empty micelles increases, a larger fraction of intermicellar collisions becomes ineffective, hindering both the fulfilment of the nucleation requirements and the incorporation of new atoms into nanoparticles. Therefore, growth by ripening is proposed to be responsible for the accumulation of free metal atoms as the film flexibility increases, explaining the behaviour of the curves after the maximum. To test this hypothesis, Fig. 9B compares the numbers of free atoms obtained with and without ripening. The results confirm that the broadening of the curves observed after the maximum, namely the slower decrease in the number of free atoms as the film flexibility increases, arises from the contribution of Ostwald ripening to particle growth.

**Fig. 9:**
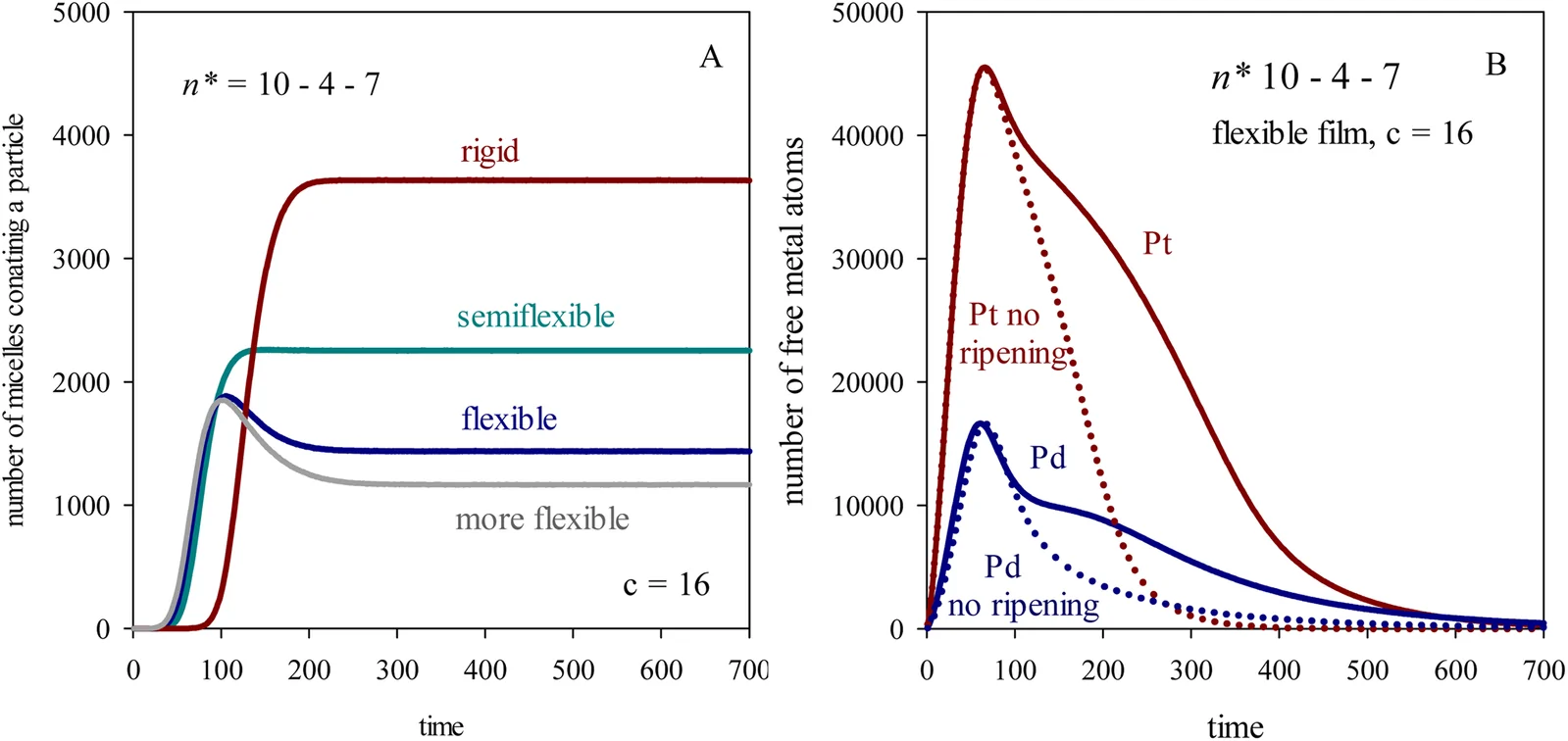
(A) Number of micelles containing a nanoparticle as a function of time. Rigid, semiflexible, flexible and more flexible films are shown in red, cyan, blue and grey, respectively. (B) Number of free atoms as a function of time. Pt is shown in red, and Pd in blue. Solid and dotted lines correspond to simulations with and without ripening, respectively. Flexible film (*f* = 30, *k*_ex_ = 5). Simulation parameters: critical nucleus sizes set n* = 10–4–7; reduction rates: *v*_Pt_ = 10%, *v*_Pd_ = 3%, 〈*c*〉 = 16 atom per micelle, 〈*c*_reductor_〉 = 10 〈*c*〉, 1 : 1 reactants ratio in feeding solution.

Finally, another point worth highlighting is that the curves representing the number of Pt and Pd atoms incorporated into nanoparticles (dash-dotted lines in Fig. 8) show that, at the beginning of the synthesis, Pt and Pd are incorporated simultaneously. This explains the composition of the nanoparticle cores (approximately 60% Pt, see Fig. 2 and 5). In other words, the critical nucleus size for pure Pt 
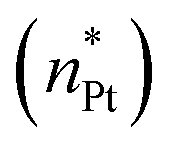
 plays essentially no role, and Pt is incorporated mainly through heteroatomic nucleation. Furthermore, the onset of Pt incorporation is delayed as the critical nucleus sizes increase, as expected. Conversely, this delay becomes shorter as the film flexibility increases, since faster intermicellar exchange allows the nucleation requirements to be satisfied more rapidly. As the synthesis progresses, faster intermicellar exchange, which reduces the influence of the critical nucleus sizes, causes the Pt and Pd deposition curves to approach each other, until the Pd curve may eventually overtake the Pt curve. A similar trend is observed as the critical nucleus sizes increase: the Pt and Pd deposition curves become progressively closer (see the dashed-dotted lines in Fig. 8, from top to bottom), and may eventually cross. This behaviour is clearly illustrated by the grey curves in Fig. 8, which represent the difference between the number of Pt and Pd atoms incorporated into the particles. This difference is positive (Pt-rich) for low critical nucleus sizes, but may become negative (Pd-rich) as the critical nucleus sizes and/or flexibility increase, giving rise to the atypical profiles shown in Fig. 5.

Summarizing, the relative amounts of Pt and Pd incorporated into the nanoparticles at each stage of the synthesis are determined by the specific combination of synthesis parameters.

### 3.5 Comparison with experimental results

Table 1 summarizes the reported one-pot synthesis of Pt/Pd nanoparticles in microemulsions under different experimental conditions (successive reduction methods are excluded), revealing distinct segregation patterns. By analyzing the synthetic parameters, the first point to note is that different metal precursors are used. In the presence of excess Cl^−^, PdCl_2_ in aqueous solution forms PdCl_4_^2−^. This is particularly important because the standard reduction potentials of the palladium chloride complex (PdCl_4_^2−^) and the uncomplexed palladium ion (Pd^2+^) are 0.646 and 0.985 V, respectively. The significantly lower reduction potential for PdCl_4_^2−^ results from the strong stabilization of Pd^2+^ by the chloride ligands.^34^ Consequently, the reduction rates of PdCl_4_^2−^ and Pd^2+^ differ. In the simulations, the effect of the precursor chemistry on the reduction kinetics is incorporated through the effective reduction-rate parameter assigned to each metal precursor. Compared with the Pt precursors (*ε*°(PtCl_6_^2−^/Pt) = 0.742 V), this leads to an opposite trend: Pd^2+^ is reduced faster than PtCl_6_^2−^, whereas PdCl_4_^2−^ is reduced more slowly. In the simulations, the reduction rate of the Pd precursor was assumed to be slower than that of the Pt precursor, corresponding to a reduction potential of the PdCl_4_^2−^/Pd couple (*ε*° = 0.646 V). Therefore, experiments (1)–(3) in Table 1 cannot be directly compared with the simulation results without adjusting the corresponding reduction–rate parameter. In these experiments, the final metal segregation can be readily explained because both the reduction and nucleation rates are higher for Pd than for Pt. This accounts for the Pt-enriched surface observed in experiment (1). The alloy structure obtained in experiment (2) may be attributed to the high surfactant film flexibility provided by the C_12_E_5_/hexadecane/water microemulsion. This high flexibility promotes faster intermicellar exchange, leading to mixing of the two metals from the early stages of nanoparticle formation. In addition, it enhances the contribution of Ostwald ripening to particle growth, further promoting metal mixing. Finally, unlike the other experiments and the simulation conditions, experiment (3) was carried out with a Pd : Pt reactants ratio 10 : 1. The large excess of Pd allows Pd atoms to reach the surface, despite its faster reduction and nucleation.

**Table 1:** Summary of the synthetic parameters reported for one pot synthesis of Pt/Pd nanoparticle in microemulsions

Exp.	Nanostructure	Precursors	Microemulsion composition	Concentration	Ref.
(1)	Surface enriched in Pt	PtCl_6_^2−^; Pd^2+^	Triton X 114 – cyclohexane *w* = 6	0.2 M	4
		Pt : Pd 1 : 1	Flexible film		
(2)	True alloy	PtCl_6_^2−^; Pd^2+^	C_12_E_5_/hexadecane/water		28
		Pt : Pd 1 : 1	Highly flexible film		
(3)	47% Pd at surface	PtCl_6_^2−^; Pd^2+^	Triton X 114 – cyclohexane *w* = 5.5	0.2 M	66
		Pd : Pt 10 : 1	Flexible film		
(4)	No core–shell, surface enriched in Pd	PtCl_6_^2−^; PdCl_4_^2−^	AOT/cyclohexane/water *w* = 6		3
		Pt : Pd 1 : 1	Rigid film		
(5)	True alloy	PtCl_6_^2−^; PdCl_4_^2−^	AOT/isooctane/water *w* = 6	0.1 M	27
		Pt : Pd 1 : 1	Rigid film		
(6)	Pt_core_/Pd_shell_	PtCl_6_^2−^; PdCl_4_^2−^	C_12_E_4_/cyclohexane/water *w* = 4	High c	29
		Pt : Pd 1 : 1	Highly flexible film		

In experiment (4), the nanoparticles do not exhibit a core–shell structure, although their surface is enriched in Pd. Since neither the reactant concentration nor the detailed microemulsion composition (required to estimate the droplet size) is reported, a direct comparison with the simulations is not possible. An AOT/cyclohexane/water microemulsion provides a rigid film, so the corresponding simulation parameters are expected to be those of a rigid (*k*_ex_ = 1, *f* = 5). Under these conditions, a no core–shell structure with a Pd-enriched surface is obtained at low reactant concentration for critical nucleus sizes of n* 10–4–7 (see the red line in Fig. 5C) and n* 10–6–8 (see the red line in Fig. 5E). In the latter case, the Pd surface enrichment is less pronounced. Higher reactant concentrations lead to core–shell structures.

Alloyed Pt/Pd nanoparticles are obtained in experiment (5) using an AOT/isooctane/water microemulsion with *w* = 6 and a reactant concentration 0.1 M. The droplet size of an AOT/isooctane/water microemulsion with *w* = 6 is 3–4 nm.^67^ Assuming a droplet radius 3.5 nm, a reactant concentration of 0.1 M corresponds to 12 reactants per droplet. The use of AOT as a surfactant results in a rigid interfacial film. Fig. 10 shows the structures obtained testing the three proposed critical nucleus size sets of under these synthesis conditions. The combination of low reactant concentration and slow intermicellar exchange makes the predicted structures highly sensitive to the critical nucleus sizes. Fig. 10A (n* 10–2–6) shows a Pd-enriched surface, whereas n* 10–4–7 set shown in Fig. 10B yields to a true alloyed structure. The n* 10–6–8 set (Fig. 10C) produces nanoparticles with a Pt-enriched surface, although the number of nanoparticles is very small. These results indicate that Fig. 10B successfully reproduces the experimental observations, suggesting that the critical nucleus size set n* 10–4–7 successful provides the most appropriate description of the Pt/Pd system. Furthermore, because cyclohexane (used in experiment (4)) provides a more rigid interface and slower intermicellar exchange rate than isooctane,^68^ the flexibility parameters for the AOT/isooctane/water system are expected to be slightly higher. Simulations performed using a semiflexible film (*k*_ex_ = 3, *f* = 15) showed the same qualitative behaviour as those obtained with a rigid film.

**Fig. 10:**
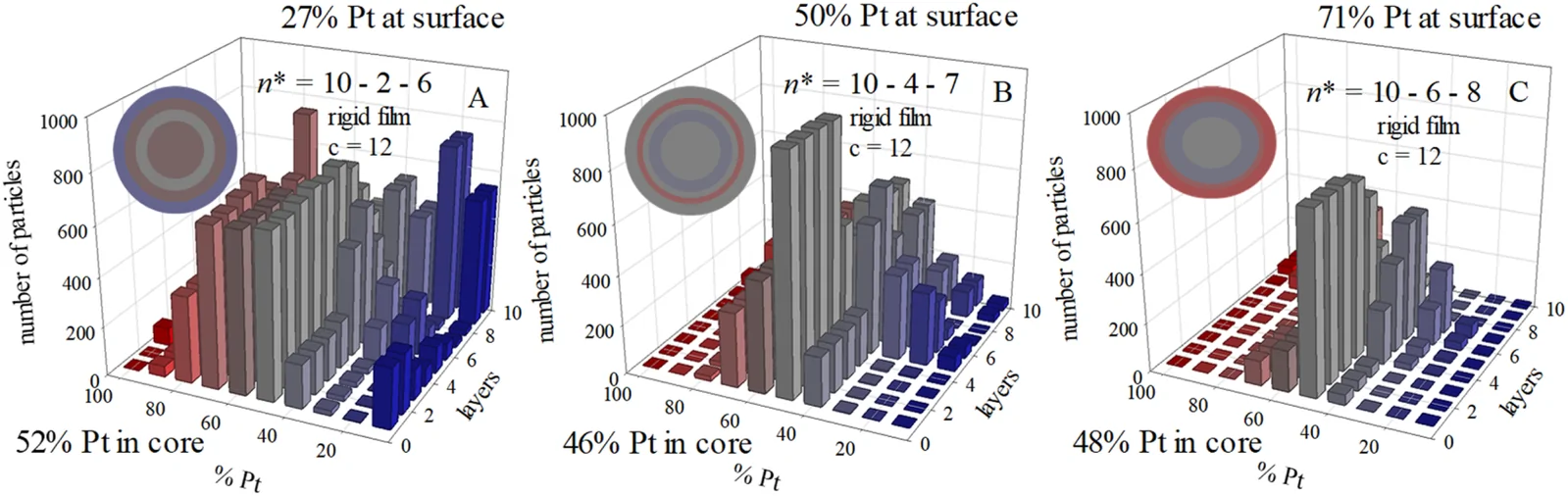
Number of particles as a function of the Pt content (%) from the core to the surface (layer by layer) for the critical nucleus size sets (A) n* 10–2–6; (B) n* 10–4–7; (C) n* 10–6–8. Colour code: blue (0–45% of Pt), grey (45–55% of Pt), red (55–100% of Pt), with lower red intensity indicating lower Pt content. Circles in each histogram represent nanoparticle structure in concentric layers using the same color scheme. Simulation parameters: reduction rates *v*_Pt_ = 10%, *v*_Pd_ = 3% reaction per collision; average reactant concentration 〈*c*〉 = 12 reactants per micelle; 〈*c*_reductor_〉 = 10 〈*c*〉 rigid film (*k*_ex_ = 1, *f* = 5).

In experiment (6), the nanoparticles exhibit a Pt core–Pd shell structure. The microemulsion consists of a C_12_E_4_/cyclohexane/water system, which provides a highly flexible interfacial film. Although the reactant concentration is not reported, the large final nanoparticle size (20–35 nm) suggests that a high reactant concentration was used. Under these conditions (high concentration and highly flexible film) the simulations predict core–shell structures for all three proposed critical nucleus size sets (see Fig. 5), in agreement with the experimental observations.

## 4 Conclusions

This work demonstrates that the architecture of Pt–Pd nanoparticles synthesized in microemulsions by the one-pot method is governed by the interplay between intermicellar exchange, reduction kinetics, nucleation kinetics, and Ostwald ripening.

Among the nucleation parameters, the critical nucleus size for heteroatomic nucleation was identified as the main factor controlling metal segregation, particularly at low reactant concentrations, where nucleation barriers play the dominant role. Increasing the reactant concentration reduces the influence of these nucleation barriers by increasing the availability of metal atoms for nucleation, leading to Pt-core/Pd-shell structures. In contrast, low reactant concentrations favour alloyed or partially segregated nanoparticles. This result is in agreement with previous simulation and experimental studies on the influence of reactant concentration on metal segregation in Au–Pt nanoparticles synthesized in microemulsions.^25^ Surfactant film flexibility has little influence on the nanoparticle core but strongly affects the surface composition by modulating the contribution of Ostwald ripening to particle growth, with higher flexibility promoting Pt surface enrichment.

Beyond providing mechanistic insight, the model establishes practical guidelines for the synthesis of Pt–Pd nanoparticles in microemulsions. For the Pt–Pd system investigated here, high reactant concentrations should be used to obtain Pt-core/Pd-shell structures, whereas lower concentrations favour alloyed or partially segregated nanoparticles. Rigid surfactant films favour Pd-enriched surfaces, while more flexible films enhance Pt surface enrichment through Ostwald ripening. Finally, the choice of metal precursors is a key design parameter because their relative reduction rates strongly influence the final elemental distribution.

The model successfully rationalizes the wide variety of Pt–Pd nanostructures reported experimentally and provides a predictive framework for tailoring both the internal architecture and the surface composition of Pt–Pd nanoparticles through the appropriate selection of metal precursors, reactant concentration, and microemulsion composition. These findings provide practical guidelines for the rational design of Pt–Pd nanocatalysts with targeted structural and surface characteristics.

## Author contributions

C. Tojo: conceptualization, methodology, visualization, simulation, writing, revision.

## Conflicts of interest

There are no conflicts to declare.

## Abbreviations

PtCl_6_^2−^: Metal precursor
PdCl_4_^2−^: Metal precursor
Pt: Reduction product of precursor PtCl_6_^2−^
Pd: Reduction product of precursor PdCl_4_^2^
R: Reducing agent
〈*c*〉: Mean number of the metal precursors (PtCl_6_^2−^or PdCl_4_^2^) per micelle at the beginning
〈*c*_R_〉: Mean number of the reductor molecules per micelle at the beginning
*k*_ex_
: Exchange rate of content in microemulsion droplets, defined as the number of free units (PtCl_6_^2−^, PdCl_4_^2−^, Pt, Pd, R) which could be transferred during a collision
*f*
: Maximum particle size for transfer between droplets (number of Pt/Pd atoms) for interdroplet transfer determining microemulsion flexibility
*ε*°: Standard reduction potential (V)
*v*_Pt_
: Percentage of PtCl_6_^2−^ within the colliding micelles which gives rise to products
*v*_Pd_
: Percentage of PdCl_4_^2^ within the colliding micelles which gives rise to products

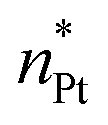

Minimum number of Pt atoms needed to form a stable nucleus

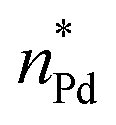

Minimum number of Pd atoms needed to form a stable nucleus

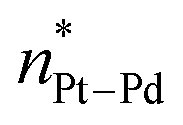

Minimum number of Pt and Pd atoms needed to form a stable nucleus
*w*
: water swelling grade (*w* = [H_2_O]/[surfactant])

## Supplementary Material

RA-OLF-D6RA06921E-s001

## Data Availability

Data is provided within the manuscript. Supplementary information (SI) is available. See DOI: https://doi.org/10.1039/d6ra06921e.
