# Supplementary material for: Tuning metal segregation and surface composition of Pt–Pd nanoparticles in microemulsions: the role of nucleation kinetics
Source: RSC Adv. 2026 Sep 29;16(45):58370–85. doi: 10.1039/d6ra06921e (PMC13621352; doi:10.1039/d6ra06921e)
Supplement: Supplementary file 1 [file RA-016-D6RA06921E-s001.pdf]

This journal is © The Royal Society of Chemistry 2026

Supporting information

A sensitivity analysis was performed to evaluate the effect of moderate variations in the Pt and Pd reduction rates around the reference values of v_{Pt} = 10%) and v_{Pd} = 3%. The analysis was performed under three representative simulation conditions, while keeping the remaining simulation parameters unchanged within each set of conditions. The reference case is v_{Pt} = 10% and v_{Pd} = 3%.

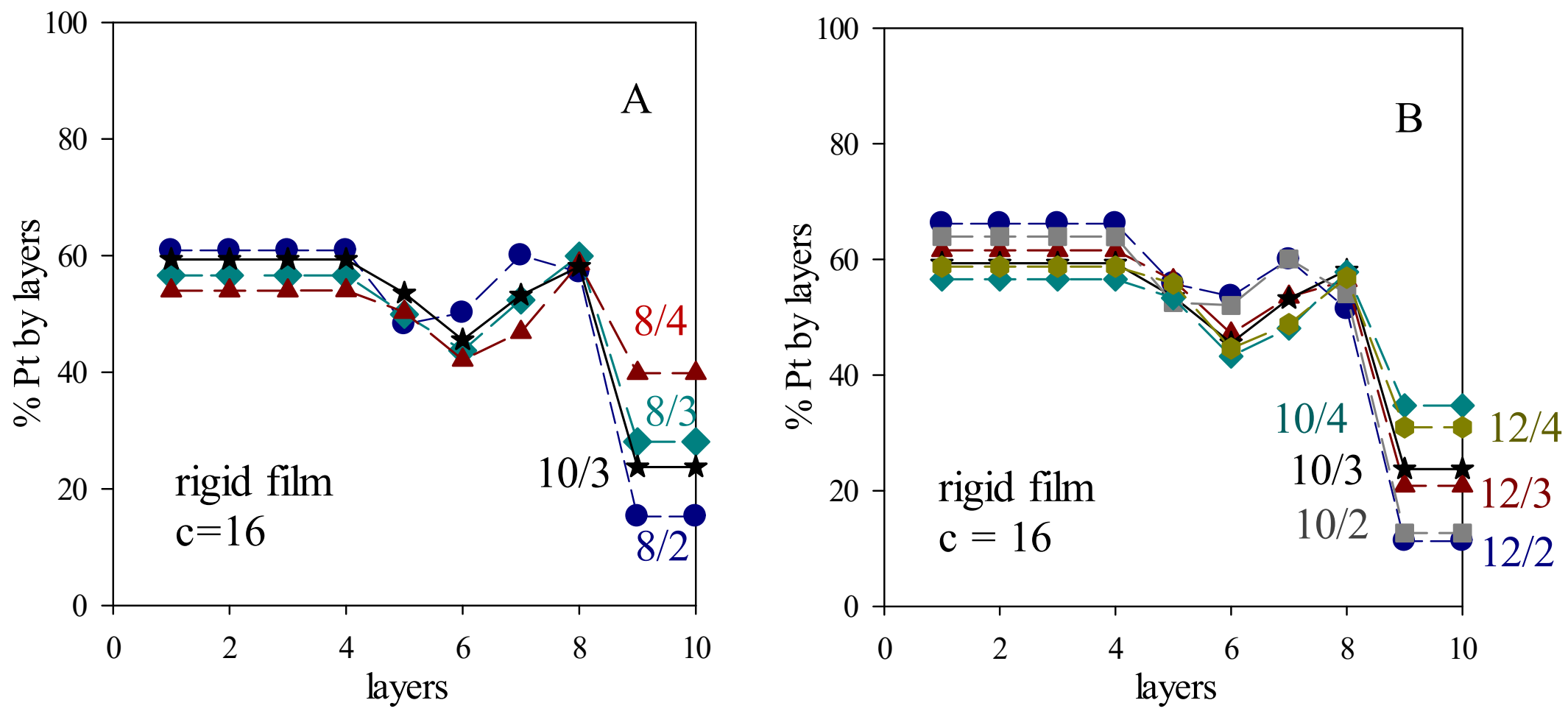

Figure S1: Average Pt content (%) as a function of layer number (1 = innermost, 10 = outermost) for the rigid film conditions, ⟨c⟩ = 16 reactants per micelle and n_{Pt} * = 10, n_{Pd}* = 4, $n_{Pt\text{-}Pd}$* =7. The reference reduction rate v_{Pt} = 10% and v_{Pd} = 3% (10/3) is represented in black. A) v_{Pt} = 8% and v_{Pd} = 2%, 3%, 4% (8/2, blue; 8/3, cyan; 8/4, red). B) v_{Pt} = 10%, v_{Pd} = 2%, 3%, 4% reaction per collision ((10/2, grey; 10/3, black; 10/4, cyan); v_{Pt} = 12%, v_{Pd} = 2%, 3%, 4% (12/2, blue; 12/3, red; 12/4, green).

Table S1. Sensitivity of the simulated Pt/Pd nanoparticle composition to variations in the reduction rates.

v_{Pt} (%)	v_{Pd}_ (%)	Pt in inner layer (%)	Pt in outermost layer (%)	ΔPt inner layer* (pp)	ΔPt outermost layer* (pp)
8	2	60.9	15.3	+1.6	−8.4
8	3	56.6	28.1	−2.7	+4.4
8	4	54.0	40.0	−5.3	+16.3
10	2	63.9	12.7	+4.6	−11.0
**10**	**3**	**59.3**	**23.7**	**0.0**	**0.0**
10	4	56.6	34.7	−2.7	+11.0
12	2	66.2	11.3	+6.9	−12.4
12	3	61.6	20.8	+2.3	−2.9
12	4	58.7	30.9	−0.6	+7.2

* ΔPt = difference in Pt content relative to the reference case v_{Pt}/v_{Pd} = 10/3, expressed as percentage points (pp).

The sensitivity analysis shows that the predicted internal composition is relatively robust to moderate variations in the assumed reduction rates. The Pt content in the representative inner layer ranges from 54.0 to 66.2%, compared with 59.3% for the reference case (see Table S1). In contrast, the outermost layer exhibits a more pronounced dependence on the relative Pt/Pd reduction rates, with the Pt content ranging from 11.3 to 40.0%, compared with 23.7% for the reference case. Importantly, despite these quantitative variations in surface composition, the overall segregation tendency predicted by the model is preserved. These results indicate that the assumed reference reduction rates mainly affect the quantitative Pt/Pd

distribution, particularly at the nanoparticle surface, but do not qualitatively determine the predicted nanoparticle architecture.

At higher metal concentrations and under more flexible-film conditions, the composition profiles obtained with different reduction rates become more similar (see Fig. S2 and S3), indicating that the influence of the reduction-rate parameters is partially attenuated under these conditions.

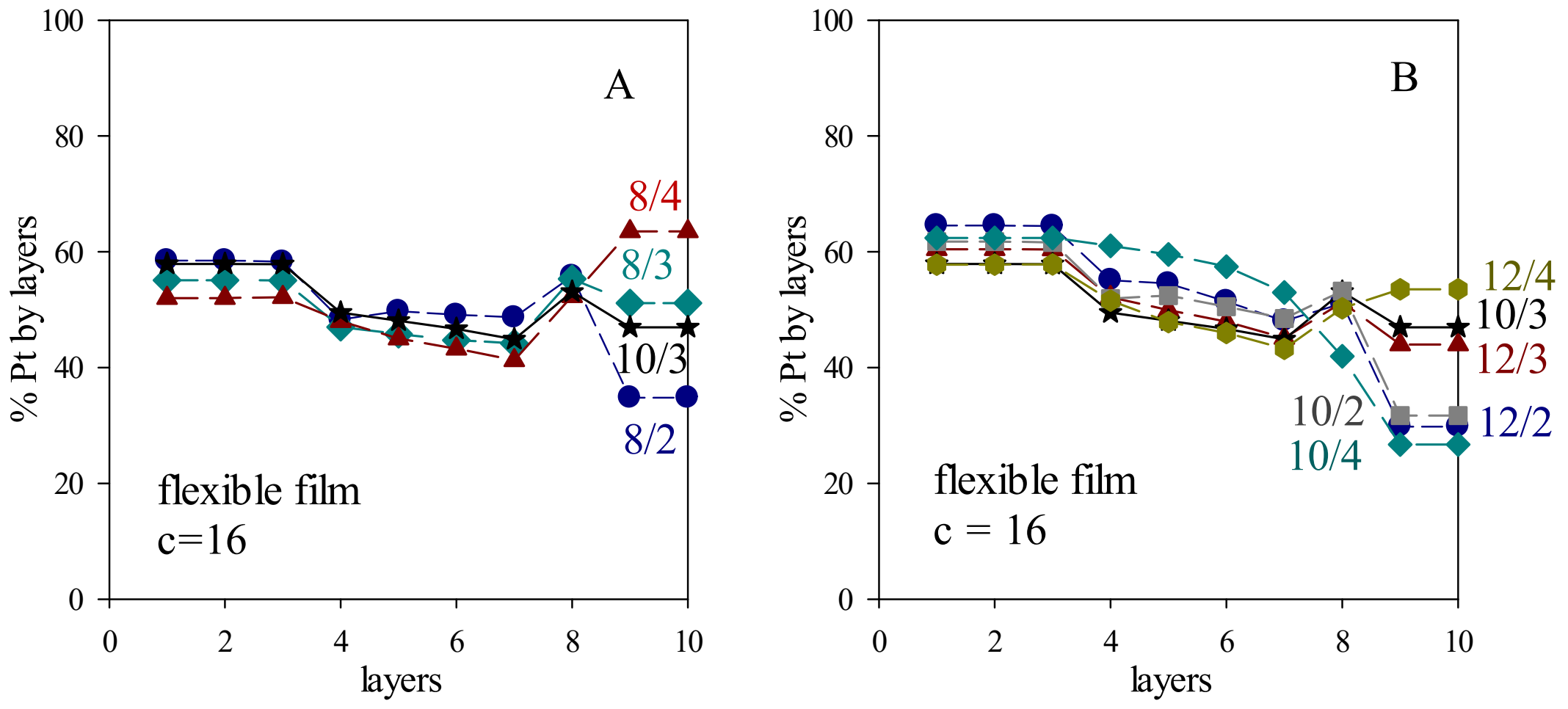

Figure S2: Average Pt content (%) as a function of layer number (1 = innermost, 10 = outermost) for the flexible film conditions, ⟨c⟩ = 16 reactants per micelle and $n_{Pt}* = 10$, $n_{Pd}* = 4$, $n_{Pt\text{-}Pd}* = 7$. The reference case $v_{Pt} = 10\%$ and $v_{Pd} = 3\%$ (10/3) is represented in black. The same reduction-rate combinations as in Figure S1 were considered.

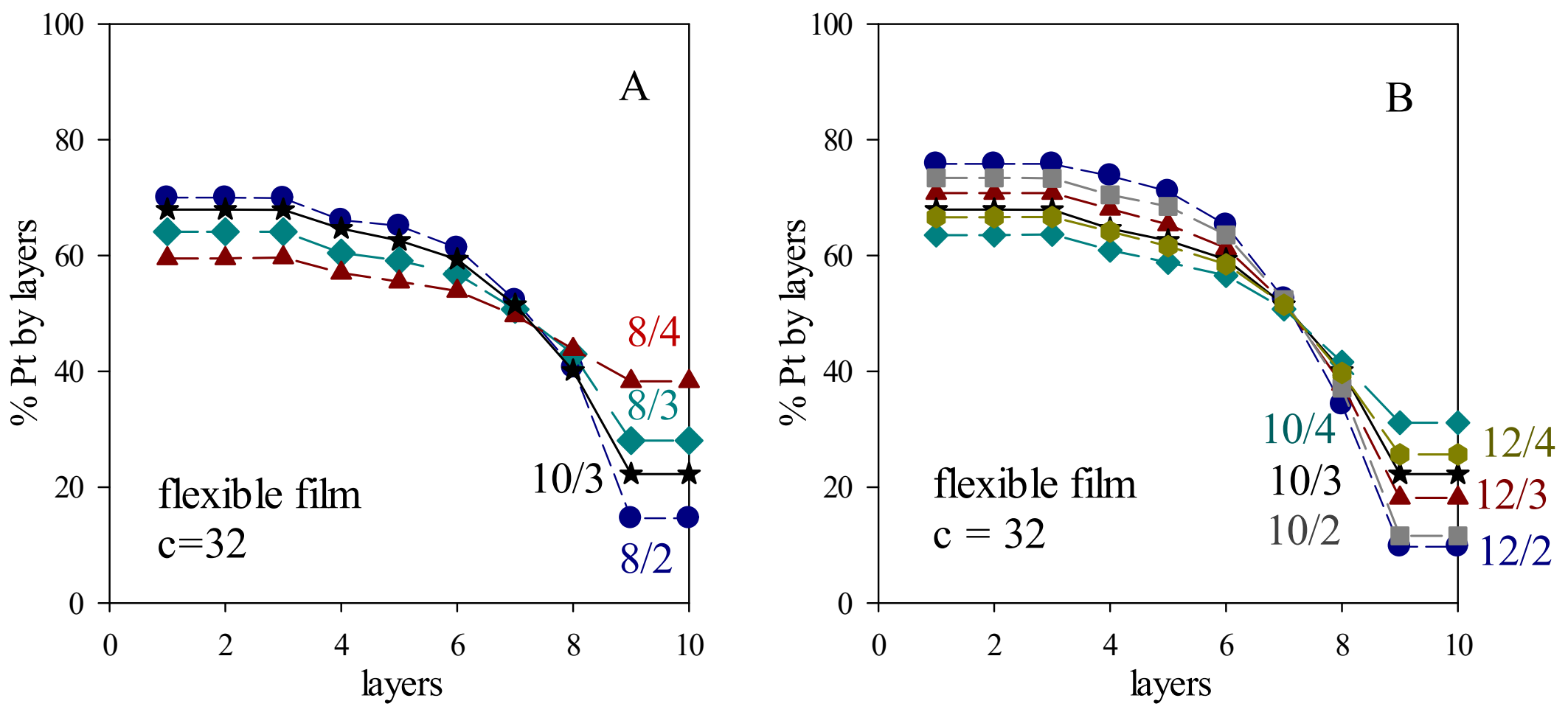

Figure S3: Average Pt content (%) as a function of layer number (1 = innermost, 10 = outermost) for the flexible film conditions, ⟨c⟩ = 32 reactants per micelle and $n_{Pt}* = 10$, $n_{Pd}* = 4$, $n_{Pt\text{-}Pd}* = 7$. The reference case is vPt = 10% and vPd = 3% (10/3) is represented in black. The same reduction-rate combinations as in Figure S1 were considered.
